# Distribution of genetic variation underlying adult migration timing in steelhead of the Columbia River basin

**DOI:** 10.1002/ece3.6641

**Published:** 2020-08-11

**Authors:** Erin E. Collins, John S. Hargrove, Thomas A. Delomas, Shawn R. Narum

**Affiliations:** ^1^ Columbia River Inter‐Tribal Fish Commission Hagerman ID USA; ^2^ Eagle Fish Genetics Lab Pacific States Marine Fisheries Commission Eagle ID USA

**Keywords:** anadromous, *greb1L*, landscape genetics, *Oncorhynchus*, population genetics

## Abstract

Fish migrations are energetically costly, especially when moving between freshwater and saltwater, but are a viable strategy for Pacific salmon and trout (*Oncorhynchus* spp.) due to the advantageous resources available at various life stages. Anadromous steelhead (*O. mykiss*) migrate vast distances and exhibit variation for adult migration phenotypes that have a genetic basis at candidate genes known as *greb1L* and *rock1*. We examined the distribution of genetic variation at 13 candidate markers spanning *greb1L*, intergenic, and *rock1* regions versus 226 neutral markers for 113 populations (*n* = 9,471) of steelhead from inland and coastal lineages in the Columbia River. Patterns of population structure with neutral markers reflected genetic similarity by geographic region as demonstrated in previous studies, but candidate markers clustered populations by genetic variation associated with adult migration timing. Mature alleles for late migration had the highest frequency overall in steelhead populations throughout the Columbia River, with only 9 of 113 populations that had a higher frequency of premature alleles for early migration. While a single haplotype block was evident for the coastal lineage, we identified multiple haplotype blocks for the inland lineage. The inland lineage had one haplotype block that corresponded to candidate markers within the *greb1L* gene and immediately upstream in the intergenic region, and the second block only contained candidate markers from the intergenic region. Haplotype frequencies had similar patterns of geographic distribution as single markers, but there were distinct differences in frequency between the two haplotype blocks for the inland lineage. This may represent multiple recombination events that differed between lineages where phenotypic differences exist between freshwater entry versus arrival timing as indicated by Micheletti et al. (2018a). Redundancy analyses were used to model environmental effects on allelic frequencies of candidate markers, and significant variables were migration distance, temperature, isothermality, and annual precipitation. This study improves our understanding of the spatial distribution of genetic variation underlying adult migration timing in steelhead as well as associated environmental factors and has direct conservation and management implications.

## Graphical abstract

Anadromous steelhead (*O. mykiss*) migrate vast distances and exhibit variation for migration phenotypes that have a genetic basis at candidate genes known as *greb1L* and *rock1*. We examined the distribution of genetic variation at 13 candidate markers spanning greb1L, intergenic, and rock1 regions versus 246 neutral markers for 113 populations (*n* = 9,471) of steelhead from inland and coastal lineages in the Columbia River. This study improves our understanding of the spatial distribution of genetic variation underlying migration timing in steelhead as well as associated environmental factors and has direct conservation and management implications.

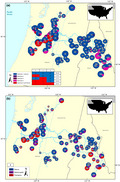

## 1 INTRODUCTION

Many animals undertake long‐distance migration from their natal sites to capitalize on abundant resources that may increase survival, fecundity, and fitness (Dingle & Drake, 2007). Migrations offer temporal and spatial availability of resources, along with seasonal suitability of migratory corridors and natal areas (Edwards & Richardson, 2004; Forrest & Miller‐Rushing, 2010). The migration of *Oncorhynchus* spp. (Pacific salmon and trout) is a critical cultural, economic, and ecological resource throughout their native range. Conservation of salmon and steelhead is based on maintaining phenotypic and genetic variation of distinct populations, and a principal focus involves conserving adult migration timings across large drainages such as the Columbia River basin. Many populations are managed according to the degree of reproductive isolation and life‐history variation. Evolutionarily significant units (ESU) of Pacific salmon and trout are defined as a distinct population segment (DPS) under the US Endangered Species Act (ESA) (Ryder, 1986; Waples, 1991) and each DPS is determined by whether it is sufficiently reproductively isolated and of evolutionary importance to the species (Waples, 1991). Since the late 1800s, wild Pacific salmon and trout have experienced a steady decline in abundance and range. The freshwater range of Pacific salmon and trout has shrunk to about 60% of the historical range (English, Peacock, & Spilsted, 2006; National Research Council, 1996). The decline has been initially attributed to overharvest, habitat degradation (logging, mining, agricultural practices), and other anthropogenic development, but modern anthropogenic activity including hydroelectric dams’ disruption of migratory routes, climate change, introgression between native populations and hatchery stocks, and an ongoing decrease in suitable habitat have also contributed to decline (Chapman, 1986; Crozier et al., 2008; Meehan, 1991).

Steelhead (*O. mykiss*) may undertake long migrations (over a thousand kilometers) in early life stages and return to natal sites to spawn (Busby et al., 1996; Keefer & Caudill, 2014). Steelhead in the Columbia River basin vary by genetic lineage that has been previously characterized as either coastal or inland (Busby et al., 1996; Quinn, 2018; Utter et al., 1980). The two genetic lineages are geographically separated: The coastal lineage inhabits streams west of the Cascade Mountains and the inland lineage inhabits streams east of the Cascades (Brannon, Powell, Quinn, & Talbot, 2004; Busby et al., 1996). Out of 15 steelhead ESUs in the Columbia River basin, 11 are listed under the ESA (Waples et al., 2001): One steelhead ESU is endangered and ten are threatened (Quinn, 2018). According to the ESA, an estimated one‐third of Pacific salmon and trout populations and all five DPS of steelhead in the Columbia River are listed as threatened or endangered (Gustafson et al., 2007). Steelhead have also been extirpated from the upper Snake River and Columbia River headwaters (Gustafson et al., 2007).

Populations of steelhead consist of individuals that spawn at similar times and are genetically similar at neutral genetic markers, but adult individuals within a population may display significant variation in when they enter freshwater or arrive at spawning grounds (Quinn, 2018). Steelhead spawn in the spring, but can begin adult migration as early as summer of the previous year before spawning or as late as winter/spring just before spawning (Quinn, McGinnity, & Reed, 2015). Steelhead adult migration may be characterized as bimodal in some rivers (Hess, Zendt, Matala, & Narum, 2016; Leider, Chilcote, & Loch, 1986), with adult migrations referred to as early migrating summer run (premature) or late migrating winter run (mature; Quinn et al., 2015). Steelhead that exhibit early migration enter freshwater before they are sexually mature, and then hold in freshwater for several months throughout the winter before maturing and spawning the following spring (Quinn, 2018; Quinn et al., 2015). Steelhead that exhibit late migrations as adults become sexually mature in the ocean before adult migration into freshwater only weeks to a few months before spawning at natal sites in the spring (Quinn, 2018; Quinn et al., 2015). Significantly more stream‐maturing steelhead populations have been extirpated than ocean‐maturing steelhead populations (Gustafson et al., 2007).

Phenotypic traits associated with migration have been demonstrated to be heritable in both juvenile and adult Pacific salmon and trout (Carlson & Seamons, 2008; Thériault, Garant, Bernatchez, & Dodson, 2007). Additionally, migration timing of adult Pacific salmon and trout has also been demonstrated to be heritable (Quinn et al., 2015; Quinn, Unwin, & Kinnison, 2000). Further, adult migration timing is associated with a genomic region of major effect in both steelhead and Chinook salmon (*O. tshawytscha*; Hess et al., 2016; Micheletti, Hess, Zendt, & Narum, 2018; Narum, Di Genova, Micheletti, & Maass, 2018; Prince et al., 2017; Thompson et al., 2019). Restriction site‐associated DNA sequencing (RADseq) studies have revealed single‐nucleotide polymorphisms (SNPs) within the *greb1L* gene region that are associated with adult migration timing in steelhead (Hess et al., 2016; Prince et al., 2017). Additional whole‐genome resequencing approaches have revealed further SNPs associated with adult migration timing and expanded the genomic region of discovered SNPs to three more candidate genes (*rock1, mib1, abhd3*, and intergenic region between *greb1L* and *rock1*; Micheletti, Hess, et al., 2018). While this genomic region of major effect may have direct conservation applications such as refining conservation units and fisheries harvest (Waples & Lindley, 2018), further understanding is needed including inheritance patterns and linkage relationships among candidate markers, and the influence of landscape characters on the distribution and frequency of candidate markers.

The *greb1L* gene is broadly present and conserved in vertebrates and the function is believed to be similar to *greb1*, which has been shown to modulate estrogen receptors and augment the role of estrogen receptor‐mediated gene expression in humans (Mohammed et al., 2013). Markers shown to have nonconservative and nonsynonymous mutations by Micheletti, Hess, et al. (2018) indicate that this genetic region is under selection and the markers in the intergenic region, upstream of *greb1L*, associated with adult migration timing could be promoters or enhancers and regulate expression of *greb1L* (Kilpinen et al., 2013). Recent studies suggest that *greb1L* plays a role in early and late adult migration phenotypes in steelhead and Chinook salmon (Hess et al., 2016; Micheletti, Hess, et al., 2018; Narum et al., 2018; Prince et al., 2017; Thompson et al., 2019). Adult migration to spawning grounds is intrinsically linked to sexual development and maturation in Pacific salmon and trout, and these processes have been attributed to *greb1L* in chum salmon (*Oncorhynchus keta*) and other species (Choi, Kim, Shin, & Choi, 2014; Ghosh, Thompson, & Weigel, 2000; Pellegrini et al., 2012; Rae et al., 2006).

In this study, we examined the distribution of genetic variation for the candidate genomic region associated with adult migration timing in steelhead to better inform conservation and management decisions across the Columbia River basin. To supplement and improve upon findings of previous studies, we were able to expand the number of candidate markers associated with adult migration timing, the number of individuals sampled, and escalate sampling coverage across the Columbia River basin (Hess et al., 2016; Micheletti, Hess, et al., 2018; Prince et al., 2017). We used 13 candidate markers spanning *greb1L*, *rock1*, and the intergenic region to test combinations of markers and identify the haplotypes most representative of adult migration timing phenotypes across a large number of steelhead populations. Four of the candidate markers were previously identified with RADseq and pooled sequencing methods (Hess et al., 2016; Micheletti, Hess, et al., 2018), and nine additional candidate markers were developed from SNPs identified with pooled sequencing methods (Table 1). Sample collections were distributed across the Columbia River basin, allowing for comparisons of candidate allelic and haplotypic frequencies for adult migration timing in a variety of steelhead habitats to better understand the spatial distribution of genetic variation underlying adult steelhead migration timing. Finally, we use landscape genetic analyses to expand upon the evaluation of environmental drivers of genetic variation identified by Micheletti, Matala, Matala, and Narum (2018) for these candidate markers and for an expansion of collection sites. To distinguish between adult migration timing phenotypes and associated genetic variation, we use the terminology of "early" and "late" to refer to adult migration phenotypes and “premature” and “mature” to refer to genetic variation (alleles, genotypes, or haplotypes).

**Table 1:** Adult steelhead migration timing‐associated candidate marker information

Order ID	SNP	Chr	Position	Gene	Forward primer	Reverse primer	Probe	Orientation
1	Omy28_11607954	28	11,607,954	*greb1L*	TGACACTGATCACAATGGTGAAAT	TAAACTGGAAGGAGAGAGCAAAAT	TGTGGGCTGC**[A/G]**AACATACTCA	+
2	Omy_RAD52458‐17	28	11,609,794	*greb1L*	ACGTGTCCCTGAGGATGGTA	AGCTCTAGGTCTGGGTCCTG	ATGGCCC**[C/A][**CT]AAGAACCC	‐
3	Omy_GREB1_05	28	11,618,027	*greb1L*	TGGGCAGATATGGAAGAACGG	ACCTTCTAAATGGCCTCTGTGT	CGGTGGCTC**[T/G]**C	+
4	Omy28_11625241	28	11,625,241	*greb1L*	CAACATTTAGGGAGAGGTTGCTAT	ATCATCAAGTTTGCCTACGACAC	CCTCCTCCCT**[A/G]**TGGTTGTCTC	+
5	Omy28_11632591	28	11,632,591	*greb1L*	GTAGAGGCCAAAGGCTTGAG	TGCTCTTATTACCTTCCAGACTCC	TGAGAA**[G/A]**AACACAGAGG	+
6	Omy_GREB1_09	28	11,641,623	*greb1L*	CCAGTGGCAACCTCAGGTAG	GACTCCAGTCACCCAAGTCA	TCAA**[T/G]**GGAGA	+
7	Omy28_11658853	28	11,658,853	intergenic	CAACATATGACCACTCGAAAACTC	ATTAATCACACCGTGAGACTCCTC	TGGTACAGAC**[A/C]**CGCACTAGCA	+
8	Omy28_11667578	28	11,667,578	intergenic	ACAGTAAACCCATTCAGGCATAGT	TTATCCTCTCAATCCACATCAAGA	GTATTGATCC**[T/C]**GTGGGAGACA	+
9	Omy_RAD47080−54	28	11,667,915	intergenic	TCAAAACCTGCAGGACTTGGA	TGGTTATATCTACAGTACAGTTCGT	TGCAAG**[A/G]**CTTAAAACGA	+
10	Omy28_11671116	28	11,671,116	intergenic	AATTTCCCCAAATTTGAAACTCTT	GTGTACATTGTCAGGCAGAAACAT	CTGGTGAGAA**[C/T]**AGGAATTACC	+
11	Omy28_11676622	28	11,676,622	intergenic	CGAATGCACTGTAGCTCATTCTAA	GCAGTAGAATGTCTCGCAAATACA	ACATGTCATT**[T/G]**ATTGTTATCT	+
12	Omy28_11683204	28	11,683,204	intergenic	CAAGAAAGAAACAGATGTTGTCCA	TTGTGACTCAAATCTGCAACCTAT	ATGTAAAAAA**[G/T]**GGCAGAAAA	+
13	Omy28_11773194	28	11,773,194	*rock1*	AGTTTGACACCCCTGTACTAGAGC	GTCTAACAAGCTCTGGGTGATTTA	GCAATTTTTT**[T/A]**AAATTACCGC	+

Note

The “Order ID” column corresponds to the SNP order, according to the physical position within the genome assembly. SNP names, chromosome number, position, gene, primers, probes, and orientation are also listed and are based on the genome assembly NCBI accession GCF_002163495.1. The premature allele is indicated in the probe column with an underline.

## 2 METHODS

### 2.1 Sample collection

**Figure 1:**
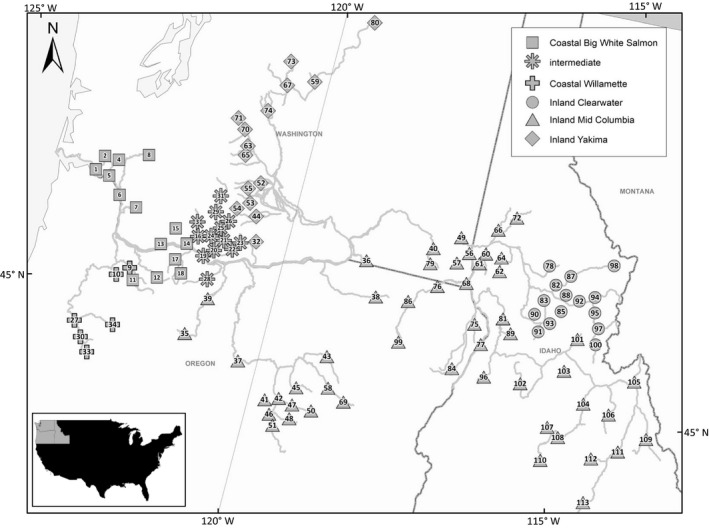
Steelhead collection sites numbered according to Table 2

Natural‐origin steelhead were collected from populations of both the inland and coastal lineages across multiple years from 1996 to 2018. Samples were collected with a variety of methods, such as electrofishing, smolt traps, and weirs. Nonlethal fin tissue samples and biologically relevant metrics were collected from both smolts and returning adults (Table S1). Steelhead were collected from locations distributed throughout the Columbia River basin with sample sizes and coordinates for each collection provided in Figure 1 and Table S1.

### 2.2 Molecular methods

DNA was extracted from tissue in accordance with a Chelex 100 method (Sigma‐Aldrich, St Louis, MO) from a total of 9,471 steelhead representing 113 collection sites and the sample size ranged between 16 and 589 steelhead from each collection (Table S1). All specimens were genotyped with genotyping‐in‐thousand by sequencing method (GTseq) as described in detail in Campbell, Harmon, and Narum (2015). Briefly, our study followed standard GTseq methods that involved two rounds of PCR to first amplify targeted SNPs and then add dual barcodes to enable each individual sample to be identified. After the dual barcoding step, the concentration of each sample was normalized and then pooled into a single tube as a “library” of samples for sequencing. Multiple libraries were prepared with ~1,000 samples per library, and between 3 and 5 libraries were sequenced on an Illumina NextSeq 550 instrument prior to genotyping with scripts from Campbell et al. (2015). All samples and loci with ≥10% missing genotypes were removed from further analyses for quality control purposes. Over the period that these individuals were genotyped, various genetic marker panel updates occurred, resulting in slight variances of the mix of putatively neutral and adaptive markers available (Table 1; Tables S1, S2). Samples were genotyped with GTseq panels ranging from 368 to 390 SNPs, and genotype data were retained when >90% loci successfully genotyped and had an estimated <0.5% genotyping error based on replicate genotyping.

### 2.3 Statistical analyses

#### 2.3.1 Population structure and genetic lineages

Putatively neutral markers were assessed using a combination of multivariate methods to detect underlying population structure, which we expected to coincide with coastal and inland lineages described in previous studies (Blankenship et al., 2011; Matala, Ackerman, Campbell, & Narum, 2014; Micheletti, Matala, et al., 2018). All neutral markers were mapped to their physical location on the *O. mykiss* genome assembly available in NCBI (accession number GCF_002163495.1), and multiple markers were found on all chromosomes with physical distance ranging from 194 KB to 39 MB. All markers had physical distance greater than 194 KB which would be greater than expected linkage decay in this species and thus were not expected to be in linkage disequilibrium. This expectation was tested with pairwise LD estimates in GenePop for a representative subsample of 25 collections. In cases where markers were consistently significant for LD tests in multiple populations, one in each significant pair was removed leaving a total of 226 markers for all subsequent analyses with neutral markers.

A principal component analysis (PCA) was plotted for all populations based on allele frequencies of putatively neutral markers determined to be without linkage disequilibrium (LD). A discriminant analysis of principal components (DAPC) was conducted with the R package adegenet 2.1.0 to assign probability of individual membership to genetic groups (*K*) (Jombart, 2008; Jombart & Ahmed, 2011). The DAPC recovers maximum genetic variation between groups, while minimizing genetic variation within groups (Jombart, 2008; Jombart & Ahmed, 2011). The adegenet package was used to identify clusters with successive *K*‐means and ran for 25 instances for *K* = 1 through *K* = 10. The Bayesian information criterion (BIC) was averaged and scaled by the standard deviation for each *K* value. The most appropriate number of genetic groups was determined with the greatest Δ*K* value as described in Evanno, Regnaut, and Goudet (2005). The LEA 2.0 R package was used to estimate population structure through sparse non‐negative matrix factorization (Frichot & François, 2015).

The distribution of genetic variation underlying adult migration timing in steelhead across the landscape was described by genotype frequencies. We examined 13 markers occurring on chromosome 28 within the *greb1L, rock1*, and intergenic region between *greb1L* and *rock1* that were previously shown to be strongly associated with adult migration timing (Hess et al., 2016; Micheletti, Hess, et al., 2018; Table 1). Initially, the two most significant SNPs were retained from a previous RAD study (Hess et al., 2016), and the remaining 11 SNPs with the strongest association with adult migration timing from the whole‐genome resequencing conducted by Micheletti, Hess, et al. (2018). To reduce ascertainment bias, we examined genetic variation in this candidate region from several populations of *O. mykiss* in the region to design primers (Table 1). Premature, mature, and heterozygote genotypes for adult migration timing were established based on genotype association from previous studies (Hess et al., 2016; Micheletti, Hess, et al., 2018), as well as using a reference collection of Skamania Hatchery steelhead, which is a hatchery strain intensively selected for early adult migration and cultured since 1956 with steelhead from the Washougal and Klickitat Rivers (Chilcote, Leider, & Loch, 1986; Crawford, 1979). Premature, mature, and heterozygote adult migration timing genotype proportions were assessed across all collection locations. A PCA of allele frequencies of adaptive markers was also conducted for all collection locations to assess genetic groupings based on adult migration timing.

#### 2.3.2 Haplotype blocks and frequencies

We assessed linkage disequilibrium (LD) within the 13 candidate markers to identify haplotype blocks that would be informative for estimating frequencies of adult migration types. Haplotype blocks within the 13 candidate markers were defined with solid spine LD analysis in the Java Runtime Environment software, Haploview 4.2, across all collection locations (Barrett, Fry, Maller, & Daly, 2005). A solid spine of LD was extended across a haploblock if D’, or a normalization of the coefficient of LD, exceeded 0.74. The same markers were assessed for LD in individuals from coastal and inland lineages (as delineated by DAPC) separately. The effect of population structure on the LD of the markers was corrected in the analysis with the LDcorSV 1.3.2 R package (Mangin et al., 2012; Table 2). Variation of genotype proportions was also evaluated with various groupings of the candidate markers.

**Table 2:** Linkage disequilibrium *R* ^2^ values corrected for population structure for all steelhead collection sites and coastal and inland sites separately

All locations	1	2	3	4	5	6	7	8	9	10	11	12
1	–	–	–	–	–	–	–	–	–	–	–	–
2	31	–	–	–	–	–	–	–	–	–	–	–
3	20	85	–	–	–	–	–	–	–	–	–	–
4	10	7	9	–	–	–	–	–	–	–	–	–
5	34	70	52	9	–	–	–	–	–	–	–	–
6	28	5	6	71	5	–	–	–	–	–	–	–
7	28	2	4	72	5	97	–	–	–	–	–	–
8	0	6	9	8	3	2	2	–	–	–	–	–
9	0	21	23	6	3	3	2	94	–	–	–	–
10	1	3	4	4	1	6	6	63	64	–	–	–
11	0	9	11	3	3	2	2	81	83	65	–	–
12	0	7	10	7	3	2	2	94	95	64	83	–
13	8	2	3	3	4	3	3	30	29	24	29	29
Coastal	**1**	**2**	**3**	**4**	**5**	**6**	**7**	**8**	**9**	**10**	**11**	**12**
1	–	–	–	–	–	–	–	–	–	–	–	–
2	70	–	–	–	–	–	–	–	–	–	–	–
3	42	72	–	–	–	–	–	–	–	–	–	–
4	8	7	10	–	–	–	–	–	–	–	–	–
5	50	68	45	5	–	–	–	–	–	–	–	–
6	20	13	17	80	3	–	–	–	–	–	–	–
7	20	4	5	83	3	99	–	–	–	–	–	–
8	3	8	10	53	4	29	30	–	–	–	–	–
9	2	16	20	37	3	48	21	81	–	–	–	–
10	19	5	5	39	3	54	54	17	25	–	–	–
11	5	12	11	16	5	10	10	57	66	25	–	–
12	3	8	11	38	4	21	22	77	85	24	63	–
13	7	5	7	11	8	8	8	9	7	2	4	7
Inland	**1**	**2**	**3**	**4**	**5**	**6**	**7**	**8**	**9**	**10**	**11**	**12**
1	–	–	–	–	–	–	–	–	–	–	–	–
2	24	–	–	–	–	–	–	–	–	–	–	–
3	19	93	–	–	–	–	–	–	–	–	–	–
4	0	3	0	–	–	–	–	–	–	–	–	–
5	27	67	56	5	–	–	–	–	–	–	–	–
6	7	1	1	47	0	–	–	–	–	–	–	–
7	7	0	0	55	0	92	–	–	–	–	–	–
8	1	7	6	0	3	1	1	–	–	–	–	–
9	1	27	26	0	4	0	1	98	–	–	–	–
10	0	1	1	0	0	0	0	76	75	–	–	–
11	1	5	7	0	2	1	1	92	91	76	–	–
12	0	9	7	0	5	1	1	96	96	73	89	–
13	2	3	4	0	5	0	0	48	47	41	52	47

Note

Column and row numbers indicate the SNP order ID from Table 1.

#### 2.3.3 Environmental influence on adaptation

Redundancy analyses (RDAs) were conducted for all Columbia River basin collections to model the degree to which the variation in environmental variables explained the variation in allele frequencies of candidate markers included in the haplotype blocks (Borcard, Legendre, & Drapeau, 1992; Kierepka & Latch, 2015). Redundancy analysis was performed on two sets of collections, all populations and each lineage (coastal versus inland), using the R package Vegan 2.5‐6 (Oksanen et al., 2019). We selected environmental variables for RDAs at collection sites in this study based on the variables significantly associated with adaptive genetic variation in a previous study (Micheletti, Matala, et al., 2018; Table 3; Table S3). When two highly correlated (>0.75 pairwise correlation; Asuero, Sayago, & Gonzalez, 2006) environmental variables were identified, one was removed from further analyses and the variable kept was determined from biological relevance to salmonids according to previous studies (Hecht, Matala, Hess, & Narum, 2015; Micheletti, Matala, et al., 2018; Olsen et al., 2011). One‐way analysis of variance (ANOVA) with Tukey's range test (Tukey, 1949) identified significant variability in salmonid habitat. Environmental variables were analyzed with the “envfit” PCA function of the vegan R package. The ANOVA test and PCA together determined significant environmental variables within and among *O. mykiss* habitats measured in this study. The final RDAs were run with significant environmental variables retained from permutation tests with 1,000 permutations (α = 0.05). Frequency of alleles in the haplotype block associated with adult migration timing was correlated with environmental variables with RDA constraint scores. Constraint scores indicated the degree of correlation and whether there was a positive or negative relationship between environmental variables and allelic frequencies.

**Table 3:** Notation, descriptions, units, resolution, variable class, source, and whether the variable was retained in the model are listed for all environmental variables assessed with the RDA models

Notation	Description	Unit	Res. (m)	Class	Source	Retained in model
mig_dist	Migration Distance	km	30	Topography	USGS	Y
elev_mean	Elevation	m	30	Topography	USGS	N
wtemp	Water Temp	°C	30	Temperature	NorWeST	Y
hli	Heat Load Index	hli	30	Temperature	ESRI	N
B1_meanT	Annual Mean Temp	°C	1,000	Temperature	WorldClim	Y
B2_meantrange	Mean Diurnal Range	°C	1,000	Temperature	WorldClim	N
B3_isotherm	Isothermality	°C	1,000	Temperature	WorldClim	Y
B4_tseason	Temp Seasonality	°C	1,000	Temperature	WorldClim	N
B5_maxtwarmmon	Max Temp Warmest Month	°C	1,000	Temperature	WorldClim	Y
B6_mintcoldmon	Min Temp Coldest Month	°C	1,000	Temperature	WorldClim	N
B7_trange	Temp Annual Range	°C	1,000	Temperature	WorldClim	N
B8_meantwetq	Mean Temp Wettest Quarter	°C	1,000	Temperature	WorldClim	N
B9_meantdryq	Mean Temp Driest Quarter	°C	1,000	Temperature	WorldClim	N
B10_meantwarmq	Mean Temp Warmest Quarter	°C	1,000	Temperature	WorldClim	N
B11_meantcoldq	Mean Temp Coldest Quarter	°C	1,000	Temperature	WorldClim	Y
B12_Prec	Annual Precip	mm	1,000	Precipitation	WorldClim	Y
B13_precwetmon	Precip Wettest Month	mm	1,000	Precipitation	WorldClim	Y
B14_precdrymon	Precip Driest Month	mm	1,000	Precipitation	WorldClim	N
B15_precseason	Precip Seasonality	mm	1,000	Precipitation	WorldClim	N
B16_precwetq	Precip Wettest Quarter	mm	1,000	Precipitation	WorldClim	N
B17_precdryq	Precip Driest Quarter	mm	1,000	Precipitation	WorldClim	N
B18_precwarmq	Precip Warmest Quarter	mm	1,000	Precipitation	WorldClim	N
B19_preccoldq	Precip Coldest Quarter	mm	1,000	Precipitation	WorldClim	N

## 3 RESULTS

After aligning markers in common for all samples and accounting for LD, 226 neutral markers (Table S2; Hess et al., 2016) and up to 13 candidate markers from chromosome 28 (Table 1) were included for further analyses. A total of 9,471 individuals from 113 populations met inclusion criteria (>90% loci successfully genotyped and had an estimated <0.5% genotyping error based on replicate genotyping) and were included in this study.

### 3.1 Population structure and genetic lineages

**Figure 2:**
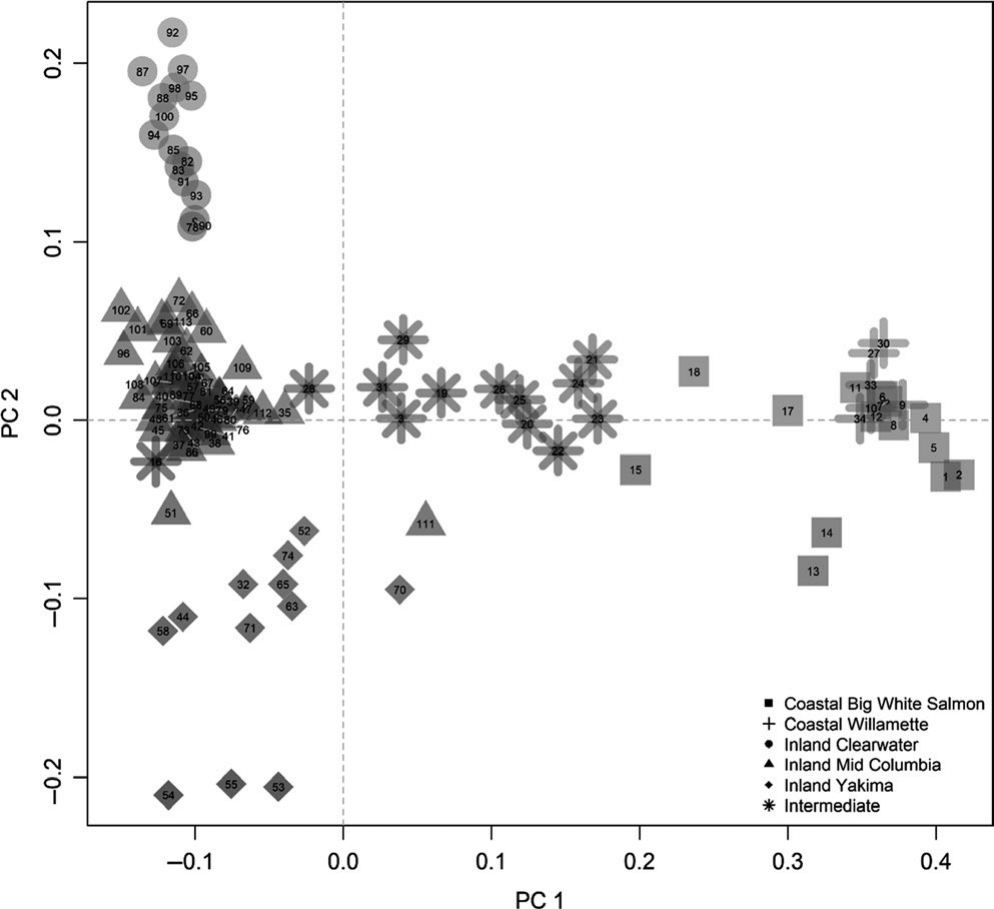
Neutral marker PCA plot for all steelhead populations. See Table 2 for collection names. Shapes indicate the geographic region of the population

**Figure 3:**
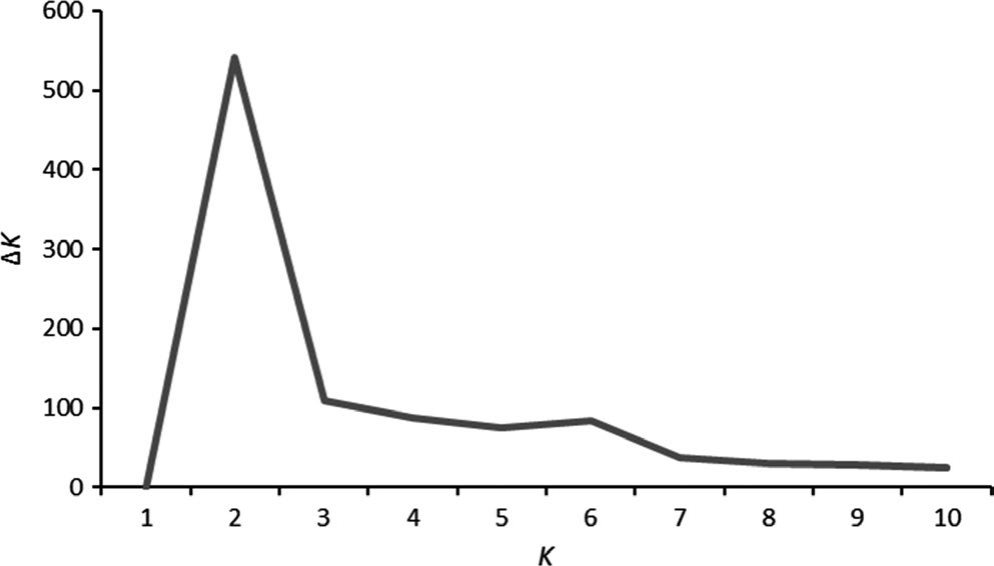
Delta *K* results based on DAPC Bayesian information criterion (BIC) values averaged over 25 iterations and divided by the standard deviation for* K* values 1–10

**Figure 4:**
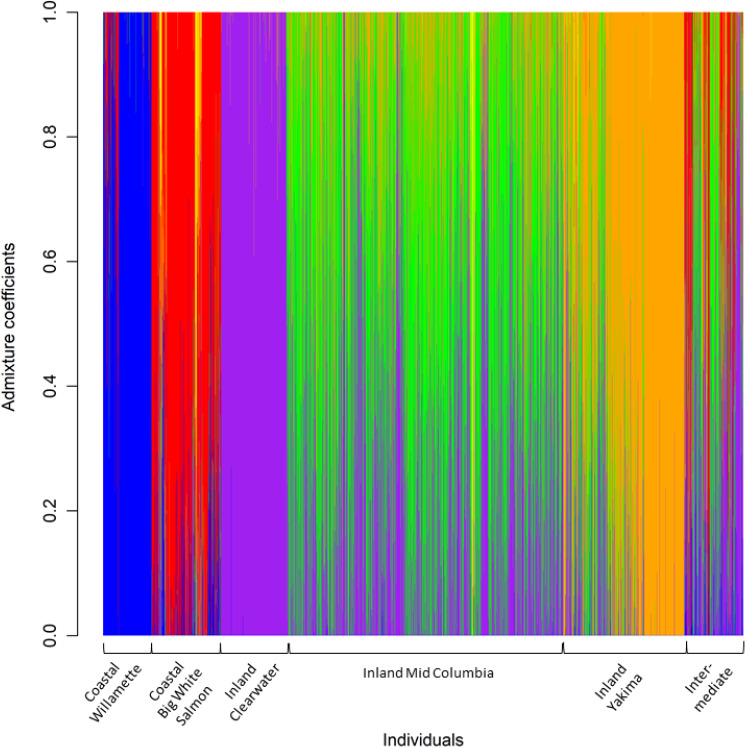
Admixture coefficients for each individual based on sparse non‐negative matrix factorization least‐squares optimizations to estimate hierarchical population structure at *K* = 6 for steelhead collections

Population structure as visualized by PCA of allelic frequencies of neutral markers indicated genetic divergence by geographic locations (Figure 2). The DAPC with neutral markers assigned steelhead to two clusters (*K* = 2): 25 putative coastal collections grouped into one cluster and 90 putative inland collections grouped into the second cluster (Figure 3). Additionally, DAPC and Δ*K* exposed hierarchical structure with a smaller peak at *K* = 6 (Figure 3). The hierarchical population structure includes well‐known population structure within the coastal and inland regions (Blankenship et al., 2011; Matala et al., 2014; Micheletti, Matala, et al., 2018), and admixture coefficient analyses were plotted for *K* = 6 with LEA to visualize the genetic mixing within finer‐scale geographic groupings (Figure 4). These finer‐scale geographic groupings are also represented by shapes in Figure 2. Most coastal collections, except for Indian Creek, exhibited nonoverlapping allele frequencies relative to all inland collections. The Klickitat River which is located between coastal and inland populations formed a cluster intermediate of the two population types. Inland collections from the Yakima and Clearwater rivers clustered distinctly from others in study (Figure 2).

**Figure 5:**
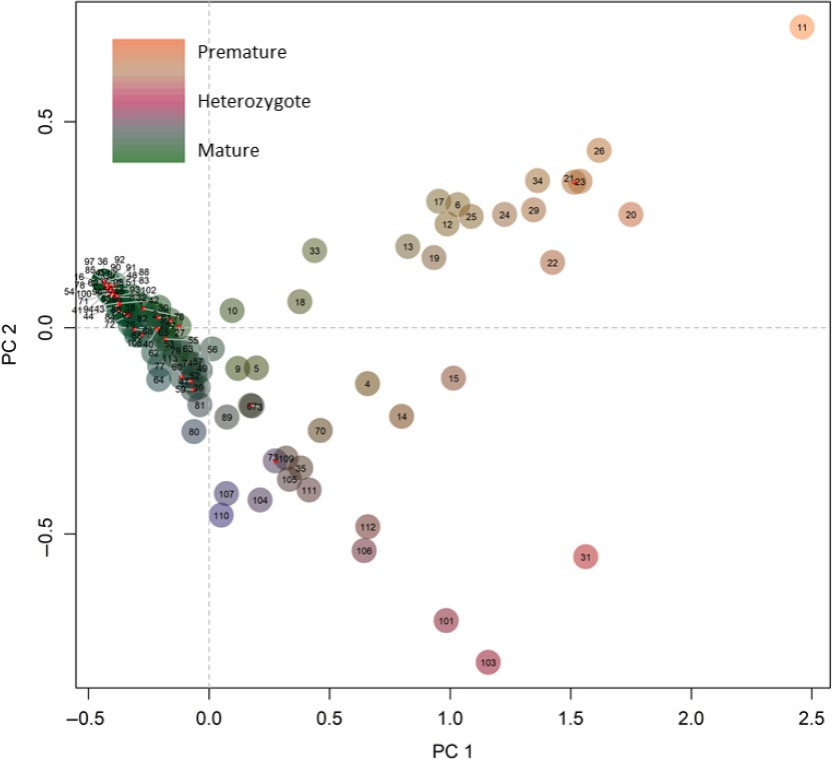
PCA of candidate markers (2, 3, 6, 9) for all steelhead populations. Populations are color‐coded by genotype (premature, mature, heterozygous) combinations of the candidate markers. See Table 2 for collection names. Four markers were included in the analysis and thus represent a range of genotype combinations shown in various shades

A second PCA was produced using candidate markers and separated individuals by the proportion of premature and mature adult migration genotypes with markers (2, 3, 6, 9) to incorporate as many collection sites as possible (Figure 5). In contrast to results with neutral markers that separated individuals by sample location and population structure, the PCA with adaptive markers separated individuals by adult migration timing genotypes.

### 3.2 Haplotype blocks and frequencies

**Figure 6:**
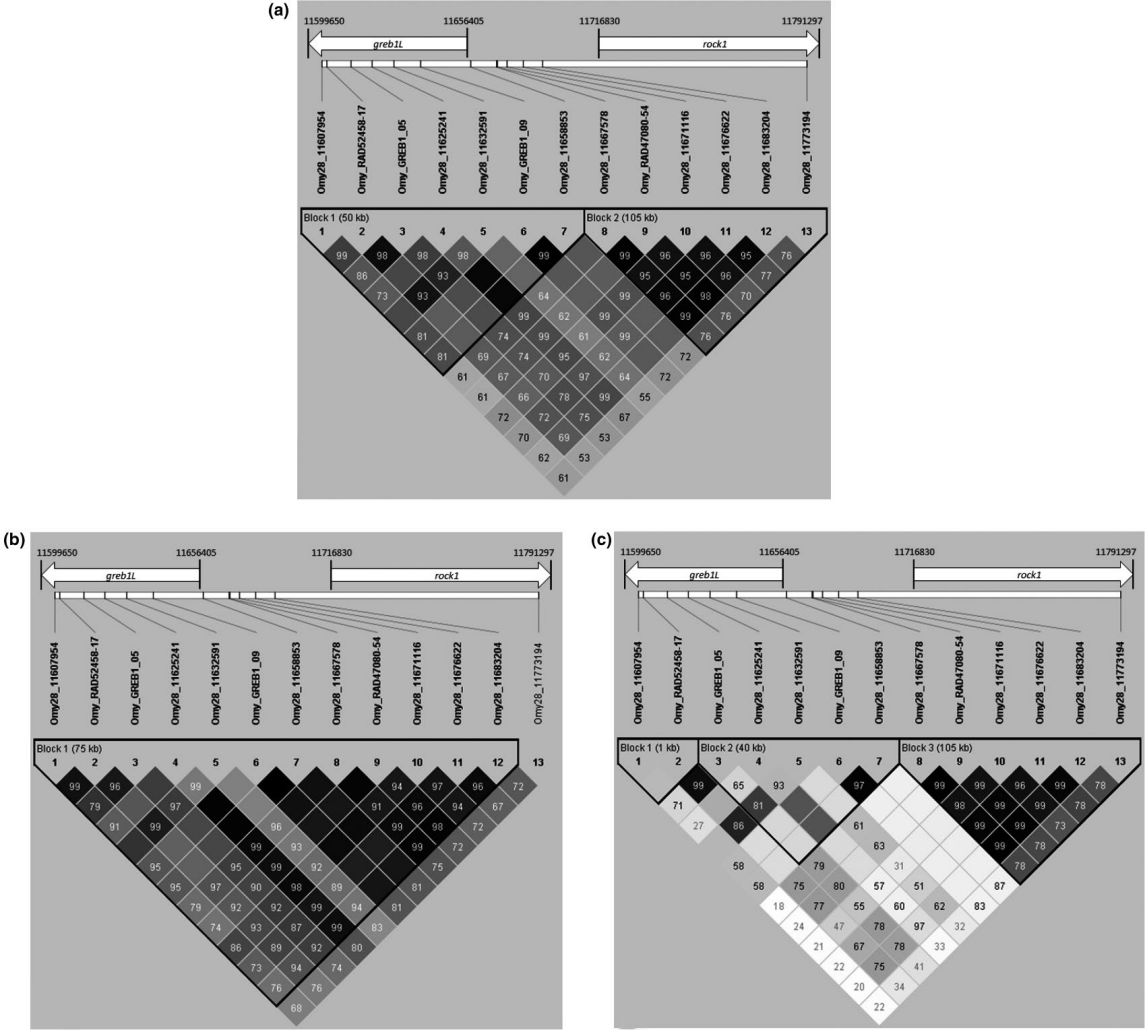
Linkage relationships for 13 candidate markers in Haploview for (a) all steelhead populations, (b) coastal populations, and (c) inland populations

Candidate markers were analyzed for all sampling locations in Haploview with solid spine, and this resulted in two haploblocks, one with markers 1–7 and another with markers 8–13 (Figure 6a). One haplotype block contained all markers within *greb1L* and another included all or the majority of markers located within the intergenic region upstream of *greb1L* and *rock1*. There was one marker located within *rock1*, but it did not demonstrate as strong of LD as other markers included in the second haplotype block. The intergenic haplotype block, containing markers 8–12, maintained high LD in both inland and coastal collections.

Haplotype blocks were examined separately for coastal (Figure 6b) and inland (Figure 6c) lineages. In the coastal lineage, high LD was retained at markers 1–12 relative to the inland lineage. Elevated LD in the coastal lineage markers resulted in one haplotype block, spanning markers 1–12 (Figure 6b). Marker 13 was not retained in the coastal lineage haplotype block and is the only marker from the *rock1* gene.

**Figure 7:**
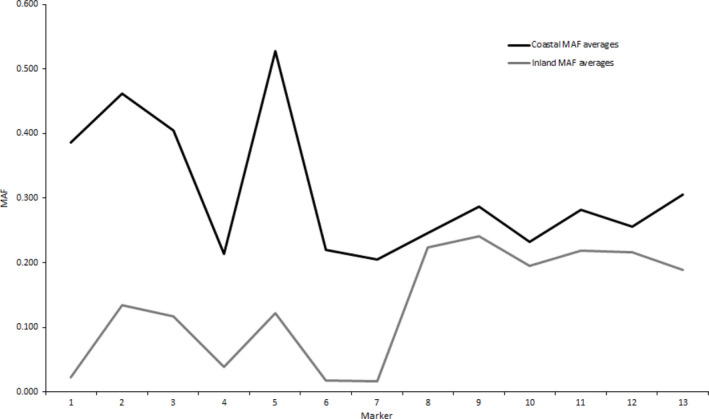
Minor allele frequency (MAF) for 13 candidate markers for each of the two major lineages of steelhead in the Columbia River. Coastal collection averages are represented by the black line, and inland collection averages are represented by the grayline

The solid spine analysis revealed three haplotype blocks in the inland lineage, which were split between markers 2 and 3 and markers 7 and 8 (Figure 6c). Additionally, minor allele frequencies (MAFs) were lower for all inland markers except for candidate markers 8–12 (Figure 7; Table S4). Variation in LD occurred among markers 1–7 and was weaker than in the coastal lineage (Figure 6b,c). The haplotype block split between markers 7 and 8 observed in the inland lineage was the same position as the split in all collections (Figure 6a), indicating the split for all collections was influenced by the inland collections. Further, a greater divergence between average MAF values can be observed between markers 7 and 8 of the inland collections than in the coastal collections (Figure 7).

Subsequent LD analyses were applied to the inland lineage samples, and variation was observed in the resulting haplotype blocks between analyses. The LD analyses done in addition to solid spine were conducted with confidence intervals (0.95 upper, 0.7 lower; Gabriel et al., 2002) and the four gamete rule, which assumes recombination when all four possible haplotypes are detected at frequencies exceeding 0.01 (frequency > 0.02–0.03; Wang, Akey, Zhang, Chakraborty, & Jin, 2002). The differing results between analyses were the inclusion or exclusion of markers 1 and 13 and the split between markers 5 and 6 or between markers 7 and 8. The difference in the location of where haplotype blocks were split could be influenced by fixed alleles at markers 4, 6, and 7 in some collections (Table S4). All Snake River haplotype block analyses were limited to markers 2, 3, 6, and 9 because these markers were developed earlier than the rest and were the only markers available when samples were collected in this basin. This resulted in limited data availability (4 instead of 13 candidate markers) for the farthest inland collections. Haploview linkage analysis comparing lineages was done both with and without the individuals that were only genotyped at 4 of the 13 markers and both analyses yielded the same results.

**Figure 8:**
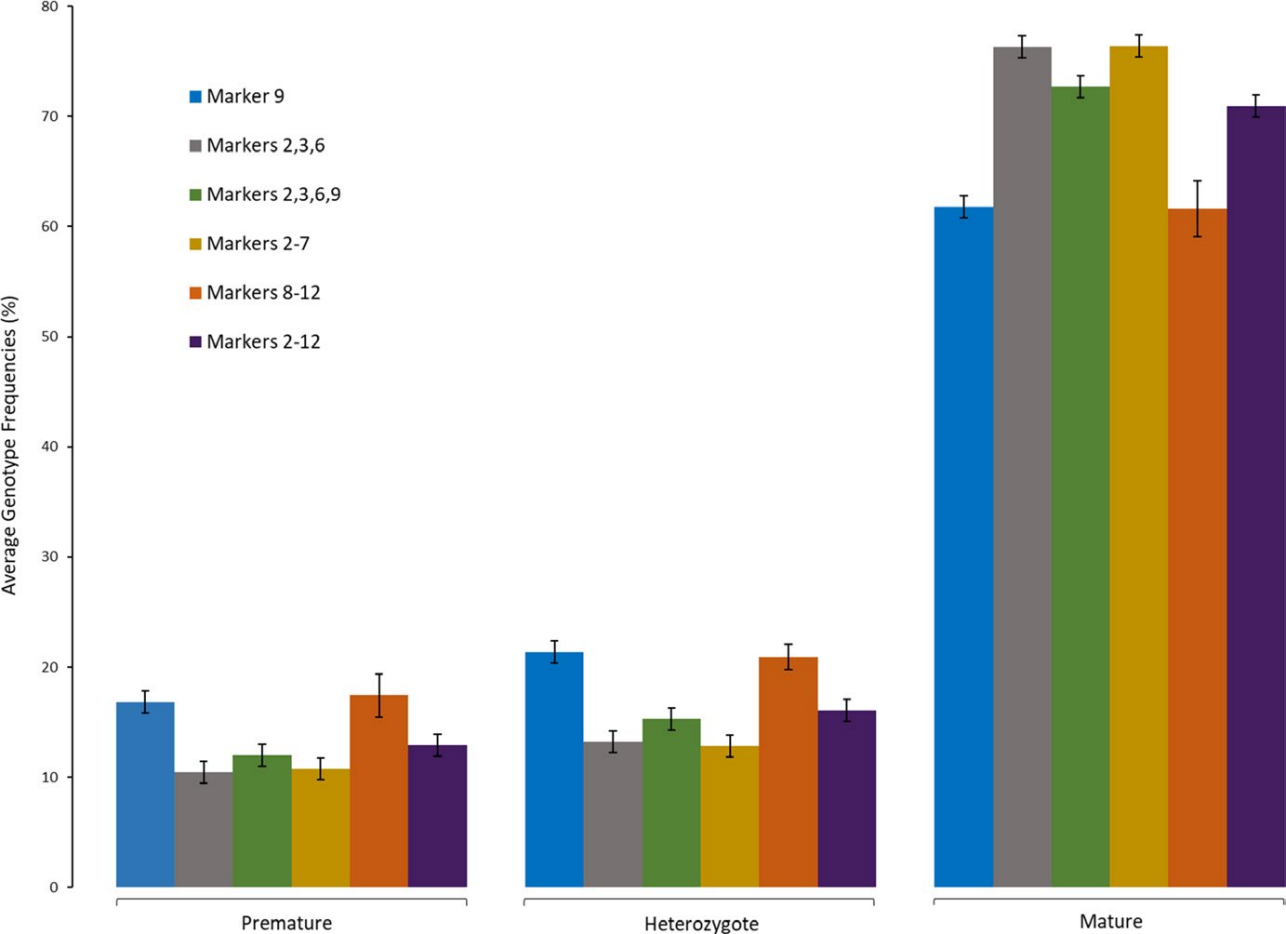
Comparison of average genotype frequencies (all steelhead populations) for different sets of candidate markers. The six sets of markers include the following: a single marker (9 as in Micheletti, Hess, et al., 2018); three markers from the same *greb1L* haplotype block (2,3,6); four markers available for all collections (2, 3, 6, 9); five markers from the intergenic haplotype block (8–12); six markers from the *greb1L* haplotype block (2–7); and 11 markers excluding one from each of the distal ends of the candidate genomic region. Error bars represent standard error

We examined six different combinations of markers to ascertain which sets of markers produce similar genotype frequencies. Genotype frequencies of marker combinations were evaluated to determine whether all markers are necessary to detect the genotypes associated with adult migration timing. The marker combinations included a single marker (9), three markers (2, 3, 6), four markers (2, 3, 6, 9), five markers (8–12), six markers (2–7), and 11 markers (2–12). This allowed for comparison across marker groups to determine whether frequencies across different marker combinations were similar. In general, all six combinations of marker groups provided similar haplotype frequencies with differences in associated haplotypes only differing by 1%–7% (Figure 8). The groups with the most similar genotype frequencies were marker 9 alone and markers 8–12; markers 2, 3, and 6 and markers 2–7 were similar; and markers 2, 3, 6, and 9 and markers 2–12 also had similar average genotype frequencies (Figure 8).

**Figure 9:**
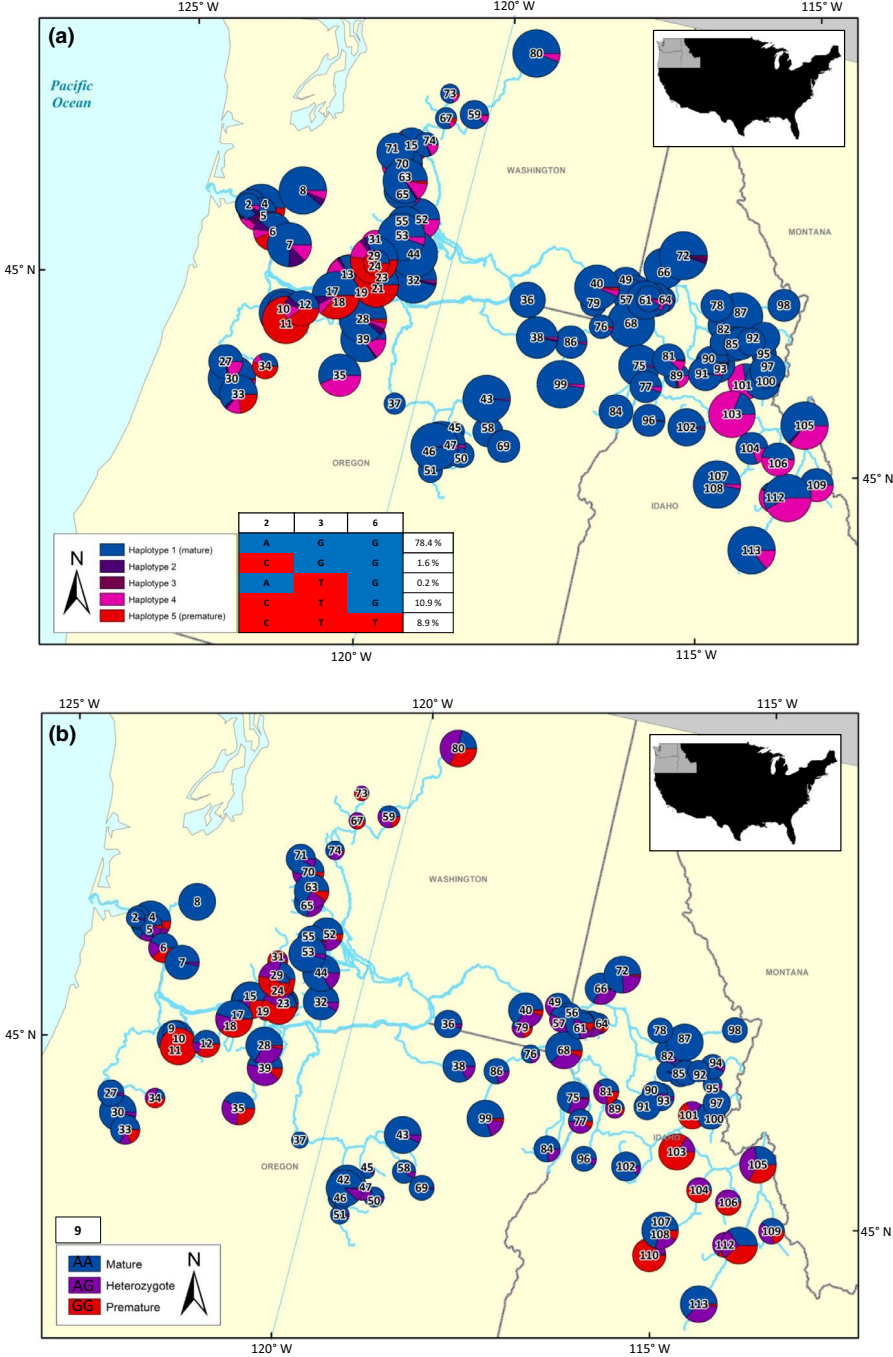
(a, b) Maps of haplotype and genotype proportions for all steelhead collection locations. Pie chart size corresponds to population size, except populations that exceeded 100 individuals were reduced to 100 with the same haplotype proportions to keep the circles on the map as visible as possible. See Table 2 for collection names and exact genotype proportions. The first map (a) demonstrates the proportions of individuals at each collection location with the five unique haplotypes from markers 2, 3, and 6. These 3 markers were evaluated to include as many populations as possible, while excluding marker 9 due to a greater association with haplotype block 2. The haplotypes representative of the heterozygote genotype are depicted as a gradient corresponding to the number of markers that match either fixed genotype. The percentage of individuals with each haplotype is reported in the table. The completely blue haplotype matches the mature genotype and is the most frequent, while the completely red haplotype matches the premature genotype and is the third most frequent. The haplotypes with a mixture of blue and red represent the different possible heterozygote genotypes. The second map (b) incorporates only candidate marker 9 (Omy_RAD47080‐54), as it was in a different linkage block than the other three markers

The mature genotype was predominant throughout much of the range in the Columbia River; however, many populations west of the Cascade Mountains and in the Salmon River have greater proportions of the premature genotype than other collections (Figure 9a,b). However, only 9 of the 113 populations had a higher frequency of premature alleles for early adult migration. To evaluate haplotype frequencies for a single haplotype block in as many locations as possible, we further scrutinized haplotypes for markers 2, 3, and 6 across the landscape and found five unique haplotypes (Figure 9a). Haplotype frequencies for collections (Figure 9a) showed similar patterns of geographic distribution as the genotype frequencies (Figure 9b), but with improved resolution for heterozygous haplotypes that were within a single haplotype block underlying *greb1L*. According to results of overall haplotype frequency (Figure 9a), the recombinant haplotype 4 is present more frequently than the premature haplotype 5. Additionally, there is a distinct separation of recombinant haplotypes between coastal (haplotypes 2 and 3) and inland (haplotype 4) collections (Figure 9a).

### 3.3 Environmental influence on adaptation

**Figure 10:**
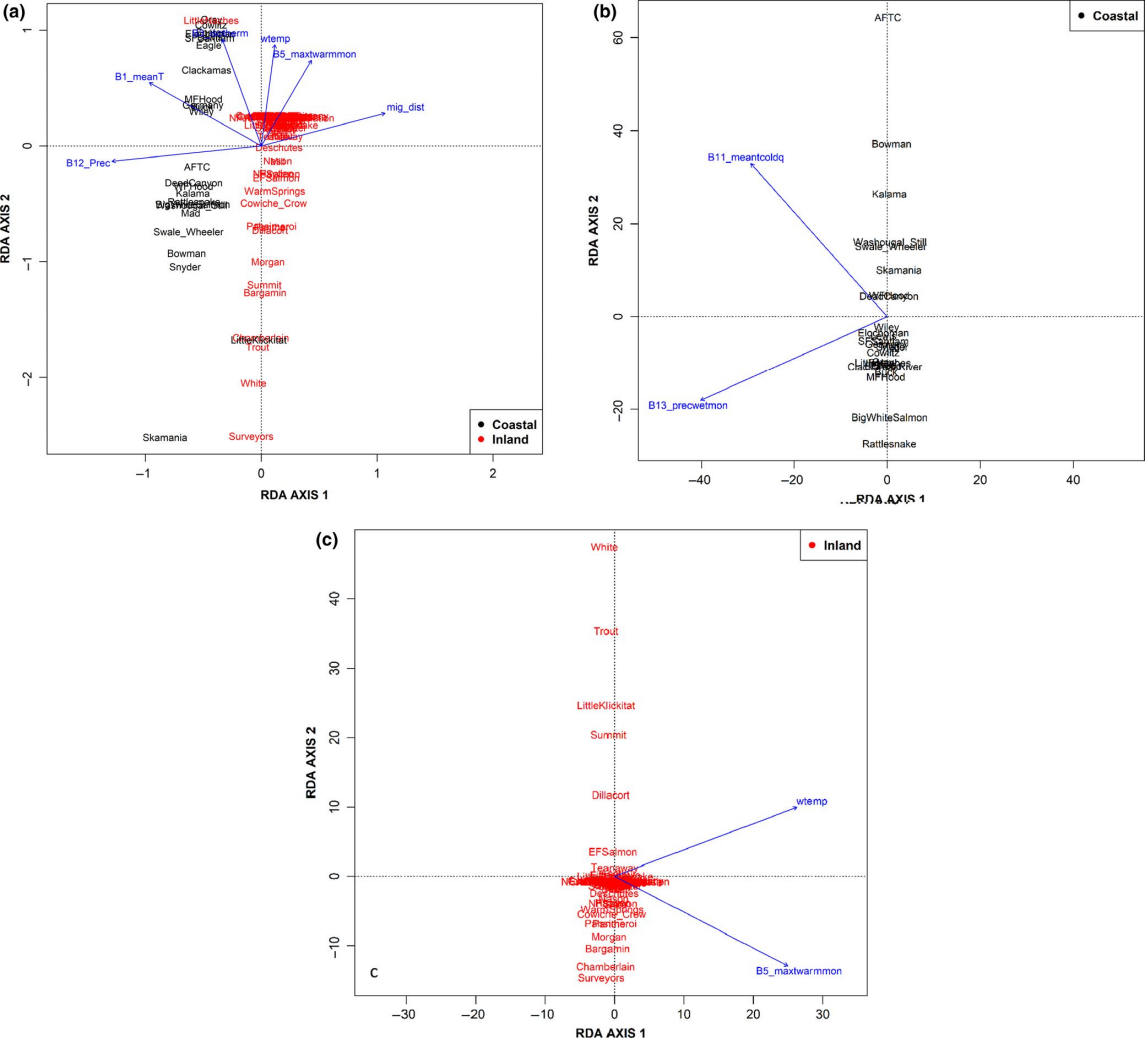
(a–c) RDA of all steelhead collections in Columbia River basin to model the degree to which the variation in environmental variables explains the variation in allele frequencies for candidate markers for all collections in the *greb1L* haplotype block (2, 3, 6). The populations are represented by text and colored black or red in accordance with their lineage determined by DAPC in adegenet. The arrows spatially denote a significant influence of environmental variables, and the length of the arrow indicates the extent of the effect. Environmental variables retained were migration distance, minimum temperature warmest month, August water temperature over a 20‐year average, annual mean temperature, isothermality, and annual precipitation. Coastal populations (b) and inland populations (c) were analyzed separately. Environmental variables retained in RDA of coastal populations were mean temperature coldest quarter and precipitation of the wettest month. Environmental variables retained in RDA of inland lineage populations were August water temperature over a 20‐year average and minimum temperature of the warmest month

To model impacts of significant environmental variables on allelic frequencies of adult migration timing‐associated markers, RDAs were done for all Columbia River basin collections and then separately for coastal and inland lineage collections. The length of the arrow from the RDA represents the magnitude of the correlation of the environmental variable in the model, and the direction of the arrow represents whether the relationship to the variable is positive or negative for a given population (Figure 10a–c). Significant environmental variables retained in the RDA for all collections were adult migration distance, minimum temperature of the warmest month, 20‐year average August water temperature, annual mean temperature, isothermality, and annual precipitation (Figure 10a). Annual precipitation had the greatest effect when all collections were analyzed together (Figure 10a). Environmental variables retained in the coastal lineage RDA were average temperature of the coldest quarter and precipitation of the wettest month (Figure 10b). Environmental variables retained in the interior lineage RDA were 20‐year average August water temperature and minimum temperature of the warmest month (Figure 10c). To compare genotypes to the environmental variables, we graphed each significant variable against the premature allele frequency for markers (2, 3, 6, 9) at each collection site (Figure S1). The relationships between genotypes and significant environmental variables were not robust for these data, but were significant for maximum temperature of the warmest month, annual precipitation, and migration distance (Figure S1).

## 4 DISCUSSION

This study provides further insight into the spatial distribution of genetic variation underlying adult migration timing in a broad range of steelhead populations. Genetic relationships were characterized for neutral markers for 113 populations, supporting previous findings of population structure and demonstrated strong differences between major lineages (Blankenship et al., 2011; Matala et al., 2014; Micheletti, Matala, et al., 2018). Further, we determined linkage blocks for 13 candidate markers associated with adult migration timing and different recombinant haplotypes were found to be predominant in coastal versus inland lineages. Environmental drivers of candidate variation revealed the importance of temperature and precipitation to selection on variation for adult migration in this system. Overall, this study provides extensive geographic variation for candidate markers associated with adult migration timing that is expected to be important for conservation applications in this species (Waples & Lindley, 2018).

### 4.1 Population structure and genetic lineages

Patterns of genetic variation among steelhead populations were highly distinct between neutral and candidate markers. Neutral structure was consistent with previous studies with various marker types that largely correspond to geographic population structure and significant heterogeneity in environmental conditions (Blankenship et al., 2011; Matala et al., 2014; Micheletti, Matala, et al., 2018). For example, steelhead in the Clearwater River have consistently shown a distinct genetic signal from others in the Snake River basin regardless of marker type (Campbell et al., 2012; Matala et al., 2014; Micheletti, Matala, et al., 2018; Narum et al., 2008). Additionally, the neutral markers provided further resolution than previous studies for the inland lineage, especially for populations in the Yakima River drainage that were distinct from the rest of the populations in the middle Columbia River. The distinct neutral patterns in the Clearwater and Yakima River drainages were likely due to different levels of genetic influence from hatchery programs (Blankenship et al., 2011). Current steelhead populations in the Yakima River are natural origin, but have been influenced by prior hatchery programs, such as introgression from Skamania and Wells stocks (Freudenthal, Lind, Visser, & Mees, 2005; Howell et al., 1985). Large stretches of the Clearwater River basin, including the Selway and Lochsa Rivers, are managed exclusively for wild fish (Campbell et al., 2012; Nielsen, Byrne, Graziano, & Kozfkay, 2009). The intermediate status of the Klickitat River collections was evident in neutral PCA clusters which are consistent with the previous studies (Micheletti, Matala, et al., 2018). This intermediate signal was also observed in two other populations, Fifteenmile Creek and Mill Creek, which may indicate gene flow with steelhead in the Klickitat River or admixture.

In contrast to geographical patterns observed at neutral loci, the candidate PCA divided collections by their predominant adult migration timing. The Skamania stock was a useful reference for the extreme extent of fixed genetic variation for premature alleles as this Skamania stock is well known for early adult migration and represents the majority of the early returning adult steelhead each migration year (Hess et al., 2016). The development of the Skamania stock started in the 1950s and included intentional selection for early returning fish so that smolts could be released within a year rather than the typical two‐year smolt age of wild fish (Crawford, 1979). At the other end of the spectrum, the mature genotype was predominant in most collections, while the heterozygote collections were dispersed across the basin, but with divergent ratios of haplotypes between coastal and inland lineages. The presence of genetic variation for premature alleles in the inland lineage suggests that some populations of steelhead (i.e., those in the Salmon River drainage) may exhibit phenotypic variation for early and late adult arrival timing to spawning grounds as shown by Micheletti, Matala, et al. (2018).

### 4.2 Haplotype blocks and frequencies

Haplotype blocks of markers with the greatest association with one another and with the adult migration timing phenotype improve ability to evaluate genetic variation associated with adult migration timing across the landscape. In addition to LD assessments, we evaluated differences between average genotype frequencies with fewer candidate markers. Marker 9 had the most similar average genotype frequencies to markers 8–12 for all genotypes, and markers 8–12 had the greatest LD in all collections. This finding suggests that marker 9 could be useful under circumstances of limited genotyping abilities. This same marker was also helpful at distinguishing patterns in steelhead arrival timing to spawning grounds as shown previously (Micheletti, Matala, et al., 2018). However, it is still beneficial to assess collections with entire haplotype blocks when possible, to generate numerous haplotype combinations instead of only three genotypes gained from a single marker.

In this study, we assessed the spatial distribution of candidate haplotype frequencies because selective pressures on adult steelhead migration are disparate across the heterogeneous landscape. The coastal lineage contained steelhead maturing both in the ocean and streams, whereas inland lineage steelhead only matured in streams. Initial studies (Hess et al., 2016; Prince et al., 2017; Thompson et al., 2019) identified and associated *greb1L* genotypes with adult freshwater entry, while Micheletti, Hess, et al. (2018) revealed a greater *greb1L* association with arrival timing to spawning grounds. We also detected more than one genotype present in inland collections, further supporting an association with arrival timing to spawning grounds because if the association was only with freshwater entry, all inland steelhead with early freshwater entry would be expected to maintain the same premature genotype. Our study incorporated more collections and more candidate markers associated with adult migration timing than previous studies, which allowed us to determine haplotypes to describe the spatial pattern of mature and premature genotypes across the Columbia River basin in greater detail. Coastal collections exhibited greater genetic diversity at candidate markers, but greater influence of premature alleles from Skamania stocks (Chilcote et al., 1986; Kostow, Marshall, & Phelps, 2003; Reisenbichler, McIntyre, Solazzi, & Landino, 1992). In the inland lineage, the mature genotype was detected at high frequency despite all inland steelhead maturing in freshwater, supporting findings by Micheletti, Hess, et al. (2018). Variation in the second haplotype block, which includes markers in the intergenic region, indicates that inland populations retain genetic variation that may allow for variable timing in arrival to spawning grounds. Additionally, the distinct separation of recombinant haplotypes between coastal (haplotypes 2 and 3) and inland (haplotype 4) collections (Figure 9a) further supports multiple recombination events within the inland lineage where phenotypic timings between freshwater entry and arrival timing differ (Micheletti, Hess, et al., 2018). However, further studies are needed that dissect arrival phenotypes and the association at candidate markers at *greb1L* and *rock1*.

### 4.3 Environmental influence on adaptation

We observed significant association between multiple environmental variables and candidate markers when examined across lineages, which was expected given that environmental conditions vary significantly across the Columbia River basin landscape. We found adult migration distances, temperature variables, and precipitation variables had the strongest association with adaptation for all collections which was consistent with previous landscape genomics analyses (Micheletti, Matala, et al., 2018). In this model, the direction of the relationship with the collection sites was not the same for each site. Significant relationships between environmental variables and candidate allele frequencies suggest that these may be environmental drivers leading to local adaptation among populations. Adult migration distance traveled between the Pacific Ocean and spawning sites ranged from 60 to 1,400 km, presenting a vast difference between coastal and inland lineages of salmonids in energetic allocation before spawning (Hecht et al., 2015; Olsen et al., 2011). However, adult migration distance was not significantly associated with candidate markers for either lineage when analyzed separately. This result suggests that variation at candidate markers is not highly distinct among populations within each of the two lineages. Significant association of temperature with candidate markers was not surprising since fish rely on environmental temperatures to regulate body temperatures and trigger migratory behavior (Jonsson, 1991; Sykes, Johnson, & Shrimpton, 2009), and extreme temperatures can inhibit cardiac and metabolic proficiencies (Chen, Farrell, Matala, Hoffman, & Narum, 2018). Further, genetic disparities in thermal tolerance when encountering temperature barriers have been found to contribute to local adaptation in salmonids (Eliason et al., 2011; Muñoz, Farrell, Heath, & Neff, 2015; Narum, Buerkle, Davey, Miller, & Hohenlohe, 2013). Finally, the significance of precipitation with variation at candidate markers is expected to be important since precipitation conditions can impact survival and selection on genes associated with thermal tolerance when stream flows are low (Heath, Busch, Kelly, & Atagi, 2002) and water temperatures are elevated (Narum et al., 2013). In contrast, when precipitation is high and stream flows are powerful, conditions may become energetically costly for migrating steelhead, but also provide cues for adult migration to spawning grounds (Keefer & Caudill, 2014; Keefer et al., 2018). Significantly associated environmental variables within each lineage were more limited than across lineages of steelhead and largely reflected regional differences in precipitation within the coastal lineage and temperature within the inland lineage.

From a management perspective, accounting for the distribution of genetic variation underlying adult migration run timing has direct conservation implications as described in detail by Waples and Lindley (2018). Early migrating fish spend less time feeding in the nutrient‐rich ocean, resulting in less opportunities for growth and potential for decreased reproductive success. Further, more time in freshwater systems exposes early migrators to thermal stress, disease, and greater risk of impacts of climate change and selective fisheries (Quinn et al., 2015). Thus, adult steelhead with this early migration pattern have increased odds of extirpation and may require greater conservation efforts (Prince et al., 2017). Previous findings (Micheletti, Hess, et al., 2018) were bolstered by this study that indicates greater genetic diversity at candidate genes for inland collections than previously understood. Effective conservation efforts to maintain or increase genetic variation underlying adult migration timing are expected to provide broader life‐history diversity for populations to endure stochastic environments. Thus, the maintenance of genetic diversity associated with adult migration timing across the Columbia River basin may be a key to promote resilient steelhead populations that are able to recover from anthropogenic impacts.

## CONFLICT OF INTEREST

None declared.

## AUTHOR CONTRIBUTION

**Erin E Collins:** Formal analysis (equal); Visualization (equal); Writing‐original draft (equal); Writing‐review & editing (equal). **John Hargrove:** Writing‐review & editing (equal). **Thomas A Delomas:** Writing‐review & editing (equal). **Shawn Narum:** Conceptualization (equal); Project administration (equal); Resources (equal); Supervision (equal); Writing‐review & editing (equal).

## Supporting information

Appendix S1

## Data Availability

Genotype data are available in Dryad at https://doi.org/10.5061/dryad.jh9w0vt80.
