# Supplementary material for: Distribution of genetic variation underlying adult migration timing in steelhead of the Columbia River basin
Source: Ecol Evol. 2020 Aug 11;10(17):9486–502. doi: 10.1002/ece3.6641 (PMC7487220; doi:10.1002/ece3.6641)

Table A1. Map identifiers, collection names, lineages, sample sizes, latitudes, longitudes, genotype proportions, year, and number of SNPs for all steelhead sample collections. The numbers in parentheses after the number of SNPs are the number of candidate markers sequenced. Lineages were determined with DAPC with markers (2,3,6,9).

| **Map ID** | **Population** | **Lineage** | **Sample size (n)** | **Latitude** | **Longitude** | **Premature genotype (%)** | **Heterozygote genotype (%)** | **Mature genotype (%)** | **Year collected** | **Number of SNPs** | **Life Stage** |
| --- | --- | --- | --- | --- | --- | --- | --- | --- | --- | --- | --- |
| 1 | Gray River | Coastal | 26 | 46.21 | -123.36 | 0.00 | 11.54 | 88.46 | 2006 | 390 (13) | Adult |
| 2 | Elochoman River | Coastal | 41 | 46.23 | -123.33 | 1.83 | 12.20 | 85.98 | 2004-2006 | 390 (13) | Adult |
| 3 | Mill Creek | Intermediate | 180 | 46.19 | -123.18 | 9.17 | 29.44 | 61.39 | 2005 | 390 (13) | Both |
| 4 | Abernathy FTC | Coastal | 90 | 46.23 | -123.15 | 22.78 | 34.44 | 42.78 | 2001-2002 | 390 (13) | Adult |
| 5 | Germany Creek | Coastal | 36 | 46.19 | -123.12 | 13.89 | 19.44 | 66.67 | 2005 | 390 (13) | Juvenile |
| 6 | Kalama River | Coastal | 64 | 46.03 | -122.87 | 39.06 | 24.61 | 36.33 | 2005 | 379 (4) | Adult |
| 7 | Lewis River | Coastal | 87 | 45.96 | -122.56 | 2.30 | 15.80 | 81.90 | 2005 | 390 (13) | Adult |
| 8 | Cowlitz River | Coastal | 120 | 46.50 | -122.59 | 0.63 | 7.29 | 92.08 | 2005 | 390 (13) | Adult |
| 9 | Eagle Creek | Coastal | 41 | 45.35 | -122.38 | 9.15 | 23.17 | 67.68 | 2005, 2006 | 390 (13) | Both |
| 10 | Clackamas River | Coastal | 194 | 45.30 | -122.35 | 8.63 | 21.13 | 70.23 | 2006, 2010 | 390 (13) | Both |
| 11 | Skamania Stock | Coastal | 301 | 45.24 | -122.28 | 100.00 | 0.00 | 0.00 | 2006 | 390 (13) | Adult |
| 12 | Washougal & Still Creeks | Coastal | 54 | 45.39 | -122.10 | 40.74 | 18.52 | 40.74 | 2006, 2010 | 390 (13) | Both |
| 13 | Buck Creek | Coastal | 72 | 45.80 | -121.53 | 20.14 | 49.31 | 30.56 | 2016, 2018 | 390 (13) | Both |
| 14 | Rattlesnake Creek | Coastal | 102 | 45.80 | -121.48 | 32.84 | 26.23 | 40.93 | 2005, 2018 | 390 (13) | Both |
| 15 | Big White Salmon | Coastal | 55 | 47.22 | -121.10 | 40.45 | 26.36 | 33.18 | 2006, 2016 | 390 (13) | Both |
| 16 | Indian Creek | Intermediate | 22 | 45.80 | -121.48 | 0.00 | 0.00 | 100.00 | 2007 | 390 (13) | Juvenile |
| 17 | WF Hood River | Coastal | 186 | 45.60 | -121.63 | 38.44 | 20.16 | 41.40 | 2008, 2011-2015 | 390 (13) | Adult |
| 18 | MF Hood River | Coastal | 42 | 45.58 | -121.63 | 13.10 | 32.14 | 54.76 | 2000 | 379 (4) | Adult |
| 19 | Dillacort Creek | Intermediate | 24 | 45.74 | -121.22 | 27.08 | 43.75 | 29.17 | 2011 | 390 (13) | Juvenile |
| 20 | Snyder Creek | Coastal | 24 | 45.82 | -121.16 | 60.42 | 34.38 | 5.21 | 2017 | 379 (4), 390 (13) | Both |
| 21 | Swale & Wheeler Creeks | Coastal | 89 | 45.81 | -121.07 | 50.56 | 35.96 | 13.48 | 2005, 2011, 2017 | 390 (13) | Both |
| 22 | Little Klickitat River | Intermediate | 70 | 45.84 | -121.06 | 50.36 | 32.50 | 17.14 | 2005 | 390 (13) | Both |
| 23 | Bowman Creek | Coastal | 82 | 45.85 | -121.04 | 52.44 | 33.84 | 13.72 | 2005 | 390 (13) | Both |
| 24 | Dead Canyon Creek | Coastal | 31 | 45.94 | -121.14 | 40.32 | 37.10 | 22.58 | 2005 | 390 (13) | Adult |
| 25 | Summit Creek | Intermediate | 52 | 45.99 | -121.13 | 35.10 | 37.02 | 27.88 | 2005 | 390 (13) | Adult |
| 26 | White Creek | Intermediate | 30 | 46.01 | -121.15 | 60.00 | 23.33 | 16.67 | 2005 | 390 (13) | Adult |
| 27 | SF Santiam River | Coastal | 51 | 44.69 | -123.01 | 2.45 | 18.14 | 79.41 | 2010 | 390 (13) | Adult |
| 28 | Fifteenmile Creek | Intermediate | 121 | 45.45 | -121.12 | 1.65 | 16.74 | 81.61 | 2012, 2013 | 390 (13) | Adult |
| 29 | Trout Creek | Intermediate | 122 | 46.04 | -121.20 | 51.64 | 22.54 | 25.82 | 2005, 2007 | 390 (13) | Adult |
| 30 | Foster | Coastal | 346 | 44.54 | -122.84 | 1.66 | 13.44 | 84.90 | 2017 | 379 (4) | Adult |
| 31 | Surveyors Creek | Intermediate | 30 | 46.20 | -121.25 | 68.33 | 15.83 | 15.83 | 2005 | 390 (13) | Adult |
| 32 | Quartz Creek | Inland | 93 | 45.95 | -120.51 | 0.00 | 4.84 | 95.16 | 2008, 2012 | 390 (13) | Both |

Table A1. Continued.

| **Map ID** | **Population** | **Lineage** | **Sample size (n)** | **Latitude** | **Longitude** | **Premature genotype (%)** | **Heterozygote genotype (%)** | **Mature genotype (%)** | **Year collected** | **Number of SNPs** | **Life Stage** |
| --- | --- | --- | --- | --- | --- | --- | --- | --- | --- | --- | --- |
| 33 | Wiley Creek | Coastal | 64 | 44.42 | -122.67 | 20.70 | 19.92 | 59.38 | 2010 | 390 (13) | Adult |
| 34 | Mad Creek | Coastal | 29 | 44.75 | -122.40 | 43.97 | 37.93 | 18.10 | 2010 | 390 (13) | Juvenile |
| 35 | Warm Springs River | Inland | 80 | 44.86 | -121.24 | 17.19 | 30.31 | 52.50 | 2008-2009 | 390 (13) | Adult |
| 36 | Touchet River | Inland | 56 | 46.03 | -118.68 | 0.00 | 1.34 | 98.66 | 2010 | 390 (13) | Adult |
| 37 | Bridge Creek | Inland | 20 | 44.73 | -120.31 | 0.00 | 0.00 | 100.00 | 2005 | 390 (13) | Juvenile |
| 38 | Iskuulpa Creek | Inland | 77 | 45.70 | -118.40 | 0.00 | 6.82 | 93.18 | 2011 | 390 (13) | Both |
| 39 | Deschutes River | Inland | 92 | 45.26 | -121.03 | 7.07 | 18.21 | 74.73 | 2011 | 390 (13) | Adult |
| 40 | Tucannon River | Inland | 89 | 46.31 | -117.66 | 1.40 | 15.17 | 83.43 | 2010, 2011, 2013 | 379 (4) | Adult |
| 41 | SF John Day River | Inland | 272 | 44.42 | -119.54 | 0.37 | 4.69 | 94.94 | 2019 | 358 (13) | Adult |
| 42 | MS John Day River | Inland | 420 | 44.46 | -119.44 | 0.24 | 6.19 | 93.57 | 2011, 2013, 2019 | 358 (13), 390 (13) | Both |
| 43 | NF John Day River & Desolation Creek | Inland | 162 | 45.00 | -118.94 | 0.46 | 2.31 | 97.22 | 2007, 2014-2016 | 358 (13), 390 (13) | Both |
| 44 | Satus Creek | Inland | 393 | 46.19 | -120.61 | 0.13 | 5.15 | 94.72 | 2017 | 379 (4) | Adult |
| 45 | Fox Creek | Inland | 16 | 44.62 | -119.29 | 0.00 | 1.56 | 98.44 | 2005 | 390 (13) | Juvenile |
| 46 | Black Canyon Creek | Inland | 43 | 44.33 | -119.57 | 0.00 | 3.49 | 96.51 | 2005, 2007 | 390 (13) | Juvenile |
| 47 | Belshaw Creek | Inland | 25 | 44.44 | -119.29 | 0.00 | 30.00 | 70.00 | 2005, 2007 | 390 (13) | Juvenile |
| 48 | Murderer's Creek | Inland | 24 | 44.32 | -119.53 | 0.00 | 0.00 | 100.00 | 2007 | 390 (13) | Both |
| 49 | Alpowa Creek | Inland | 49 | 46.41 | -117.22 | 6.63 | 19.90 | 73.47 | 2010 | 379 (4) | Adult |
| 50 | Beech Creek | Inland | 30 | 44.41 | -119.12 | 0.00 | 1.67 | 98.33 | 1996, 2005 | 390 (13) | Juvenile |
| 51 | Deer Creek | Inland | 26 | 44.19 | -119.51 | 0.00 | 0.96 | 99.04 | 2007 | 390 (13) | Juvenile |
| 52 | Ahtanum Creek | Inland | 79 | 46.53 | -120.68 | 2.53 | 25.32 | 72.15 | 2014 | 390 (13) | Both |
| 53 | Agency Creek | Inland | 28 | 46.34 | -120.87 | 2.68 | 16.07 | 81.25 | 2017 | 379 (4) | Adult |
| 54 | Toppenish Creek | Inland | 589 | 46.32 | -120.87 | 0.08 | 2.80 | 97.11 | 2009 | 390 (13) | Both |
| 55 | Simcoe Creek | Inland | 62 | 46.45 | -120.86 | 2.02 | 16.13 | 81.85 | 2017 | 379 (4) | Adult |
| 56 | Asotin Creek | Inland | 57 | 46.34 | -117.04 | 7.02 | 21.49 | 71.49 | 2010 | 379 (4) | Adult |
| 57 | George Creek | Inland | 51 | 46.30 | -117.12 | 3.92 | 22.55 | 73.53 | 2010 | 379 (4) | Adult |
| 58 | Camp Creek | Inland | 40 | 44.69 | -118.80 | 0.00 | 3.75 | 96.25 | 2005, 2007 | 390 (13) | Juvenile |
| 59 | Entiat River | Inland | 36 | 47.66 | -120.24 | 7.64 | 15.97 | 76.39 | 2006 | 379 (4), 390 (13) | Juvenile |
| 60 | Webb Creek | Inland | 30 | 46.31 | -116.81 | 4.17 | 19.17 | 76.67 | 2013 | 379 (4) | Adult |
| 61 | Sweetwater Creek | Inland | 64 | 46.29 | -116.86 | 2.34 | 12.89 | 84.77 | 2013 | 379 (4) | Adult |
| 62 | Mission Creek | Inland | 50 | 46.32 | -116.71 | 2.50 | 13.00 | 84.50 | 2013 | 379 (4) | Adult |
| 63 | Nile Creek | Inland | 88 | 46.86 | -121.05 | 5.40 | 16.76 | 77.84 | 2005, 2008, 2012 | 390 (13) | Both |
| 64 | Lapwai Creek | Inland | 32 | 46.33 | -116.60 | 3.91 | 13.28 | 82.81 | 2013 | 379 (4) | Adult |

Table A1. Continued.

| **Map ID** | **Population** | **Lineage** | **Sample size (n)** | **Latitude** | **Longitude** | **Premature genotype (%)** | **Heterozygote genotype (%)** | **Mature genotype (%)** | **Year collected** | **Number of SNPs** | **Life Stage** |
| --- | --- | --- | --- | --- | --- | --- | --- | --- | --- | --- | --- |
| 65 | Little Rattlesnake Creek | Inland | 64 | 46.76 | -121.05 | 1.17 | 17.19 | 81.64 | 2005, 2012 | 390 (13) | Both |
| 66 | Little Bear & Big Bear Creeks | Inland | 73 | 46.64 | -116.68 | 0.34 | 10.62 | 89.04 | 2012 | 379 (4) | Adult |
| 67 | Icicle Creek | Inland | 20 | 47.56 | -120.67 | 7.50 | 35.00 | 57.50 | 2007 | 390 (13) | Adult |
| 68 | Joseph Creek | Inland | 100 | 46.04 | -117.00 | 1.50 | 9.50 | 89.00 | 2011-2012 | 390 (13) | Adult |
| 69 | Clear & Granite Creeks | Inland | 46 | 44.59 | -118.51 | 0.00 | 0.54 | 99.46 | 2000, 2005, 2015 | 390 (13) | Juvenile |
| 70 | Cowiche & Crow Creeks | Inland | 73 | 47.02 | -121.17 | 15.07 | 38.36 | 46.58 | 2012 | 390 (13) | Both |
| 71 | Little Naches River | Inland | 63 | 47.11 | -121.32 | 0.00 | 2.38 | 97.62 | 2008, 2012 | 390 (13) | Both |
| 72 | Potlatch River | Inland | 102 | 46.80 | -116.42 | 0.49 | 8.33 | 91.18 | 2010, 2016 | 379 (4) | Adult |
| 73 | Nason Creek | Inland | 16 | 47.80 | -120.72 | 21.88 | 15.63 | 62.50 | 2006 | 390 (13) | Juvenile |
| 74 | Teanaway River | Inland | 26 | 47.26 | -120.88 | 3.85 | 20.19 | 75.96 | 2007, 2008 | 390 (13) | Adult |
| 75 | Lightning Creek | Inland | 76 | 45.66 | -116.73 | 0.33 | 9.21 | 90.46 | 2001 | 390 (13) | Both |
| 76 | Wenaha River | Inland | 25 | 45.95 | -117.45 | 0.00 | 13.00 | 87.00 | 2010 | 390 (13) | Juvenile |
| 77 | Sheep Creek | Inland | 44 | 45.47 | -116.56 | 2.27 | 15.91 | 81.82 | 2014 | 379 (4) | Adult |
| 78 | Lolo Creek | Inland | 46 | 46.39 | -115.72 | 0.00 | 0.00 | 100.00 | 2012 | 379 (4) | Adult |
| 79 | Crooked Creek | Inland | 31 | 46.15 | -117.65 | 5.65 | 14.52 | 79.84 | 2010 | 379 (4) | Juvenile |
| 80 | Omak Creek | Inland | 112 | 48.40 | -119.50 | 9.15 | 15.63 | 75.22 | 2010 | 390 (13) | Adult |
| 81 | White Bird Creek | Inland | 47 | 45.77 | -116.29 | 6.91 | 20.21 | 72.87 | 2014 | 379 (4) | Adult |
| 82 | Canyon Creek | Inland | 44 | 46.22 | -115.56 | 0.00 | 13.07 | 86.93 | 2004 | 379 (4) | Adult |
| 83 | Ohara Creek | Inland | 28 | 46.05 | -115.52 | 0.00 | 0.89 | 99.11 | 2013 | 379 (4) | Adult |
| 84 | Gumboot & Mahogany Creeks | Inland | 51 | 45.17 | -116.93 | 0.00 | 5.88 | 94.12 | 2011-2013 | 379 (4) | Adult |
| 85 | Gedney Creek | Inland | 51 | 46.06 | -115.31 | 0.00 | 0.00 | 100.00 | 2004 | 379 (4) | Adult |
| 86 | Grande Ronde River | Inland | 47 | 45.73 | -117.86 | 0.00 | 7.98 | 92.02 | 2011-2014 | 390 (13) | Adult |
| 87 | Fish Creek | Inland | 331 | 46.33 | -115.35 | 0.00 | 0.45 | 99.55 | 2016 | 379 (4) | Adult |
| 88 | Selway River | Inland | 47 | 46.07 | -115.24 | 0.00 | 1.60 | 98.40 | 2008 | 379 (4) | Adult |
| 89 | Slate Creek | Inland | 27 | 45.64 | -116.12 | 3.70 | 34.26 | 62.04 | 2013 | 379 (4) | Adult |
| 90 | Newsome Creek | Inland | 48 | 45.84 | -115.62 | 0.00 | 3.65 | 96.35 | 2012 | 379 (4) | Adult |
| 91 | Tenmile Creek | Inland | 51 | 45.72 | -115.68 | 0.00 | 1.96 | 98.04 | 2013 | 379 (4) | Adult |
| 92 | ThreeLinks Creek | Inland | 34 | 46.11 | -115.07 | 0.00 | 0.00 | 100.00 | 2012 | 379 (4) | Adult |
| 93 | Crooked River | Inland | 56 | 45.82 | -115.53 | 0.00 | 2.23 | 97.77 | 2013 | 379 (4) | Adult |
| 94 | Moose Creek | Inland | 44 | 46.17 | -114.89 | 0.00 | 1.70 | 98.30 | 2012 | 379 (4) | Adult |
| 95 | Bear Creek | Inland | 27 | 46.02 | -114.84 | 0.00 | 4.63 | 95.37 | 2012 | 379 (4) | Adult |
| 96 | Boulder Creek | Inland | 46 | 45.15 | -116.40 | 0.00 | 3.26 | 96.74 | 2014 | 379 (4) | Adult |

Table A1. Continued.

| **Map ID** | **Population** | **Lineage** | **Sample size (n)** | **Latitude** | **Longitude** | **Premature genotype (%)** | **Heterozygote genotype (%)** | **Mature genotype (%)** | **Year collected** | **Number of SNPs** | **Life Stage** |
| --- | --- | --- | --- | --- | --- | --- | --- | --- | --- | --- | --- |
| 97 | White Cap Creek | Inland | 55 | 45.87 | -114.73 | 0.00 | 0.00 | 100.00 | 2008 | 379 (4) | Adult |
| 98 | Crooked Fork Lochsa River | Inland | 46 | 46.53 | -114.68 | 0.00 | 0.00 | 100.00 | 2000 | 379 (4) | Adult |
| 99 | Catherine Creek | Inland | 269 | 45.31 | -117.87 | 0.84 | 6.69 | 92.47 | 2012-2014 | 390 (13) | Adult |
| 100 | Little Clearwater River | Inland | 57 | 45.75 | -114.77 | 0.00 | 4.39 | 95.61 | 2008 | 379 (4) | Adult |
| 101 | Bargamin Creek | Inland | 56 | 45.72 | -115.03 | 41.96 | 29.46 | 28.57 | 2015 | 379 (4) | Adult |
| 102 | Secesh River | Inland | 62 | 45.17 | -115.80 | 0.00 | 4.03 | 95.97 | 2011 | 379 (4) | Adult |
| 103 | Chamberlain Creek | Inland | 96 | 45.38 | -115.14 | 52.34 | 22.66 | 25.00 | 2016 | 379 (4) | Adult |
| 104 | Big Creek | Inland | 46 | 45.09 | -114.73 | 15.76 | 24.46 | 59.78 | 2011 | 379 (4) | Both |
| 105 | NF Salmon River | Inland | 217 | 44.12 | -114.43 | 16.24 | 29.95 | 53.80 | 2016 | 379 (4) | Adult |
| 106 | Panther Creek | Inland | 48 | 45.03 | -114.30 | 27.08 | 32.81 | 40.10 | 2013 | 379 (4) | Adult |
| 107 | Pistol Creek | Inland | 37 | 44.72 | -115.15 | 18.24 | 10.14 | 71.62 | 2012 | 379 (4) | Adult |
| 108 | Rapid River | Inland | 317 | 44.68 | -115.15 | 2.52 | 8.68 | 88.80 | 2012 | 379 (4) | Adult |
| 109 | Hayden Creek | Inland | 49 | 44.86 | -113.63 | 13.78 | 32.65 | 53.57 | 2017 | 379 (4) | Adult |
| 110 | Bear Valley Creek | Inland | 82 | 4.45 | -115.23 | 21.95 | 2.74 | 75.30 | 2011 | 379 (4) | Adult |
| 111 | Pahsimeroi River | Inland | 113 | 44.68 | -114.04 | 25.22 | 17.92 | 56.86 | 2016 | 379 (4) | Adult |
| 112 | Morgan Creek | Inland | 46 | 44.67 | -114.23 | 29.35 | 27.72 | 42.93 | 2012 | 379 (4) | Adult |
| 113 | EF Salmon River | Inland | 109 | 44.12 | -114.43 | 1.83 | 20.18 | 77.98 | 2009, 2010, 2016 | 379 (4) | Adult |

Table A2. Steelhead neutral marker names, chromosomes, positions, primers, probe, and orientation of the SNP based on the genome assembly NCBI accession GCF_002163495.1.

| **Locus** | **Chr** | **Position** | **Forward primer** | **Reverse primer** | **Probe** | **Orientation** |
| --- | --- | --- | --- | --- | --- | --- |
| OMS00078 | 1 | 10499333 | GAGGGAAGCAGCCATAAACAGAATA | GTCTCACTATGGTCCATATCTGTGTAGA | TCACATGCAT[A,G]AGAGTG | + |
| Omy_gadd45-332 | 1 | 12240053 | AGAGAAGACTCACTGCTGTTTGC | AAATCAGTTCCCACGCTATGCT | TTGCTCC[A,G]AAATGG | + |
| Omy_107031-704 | 1 | 18131799 | GGCTTTCGGATACTGAGCAACAA | TGAACTCACTGTTGGTATGGACTAGA | TGGACATGATT[G,A]CATAGAC | - |
| Omy_metA-161 | 1 | 24257340 | CGCATGCACCAGTTGTAAGAAAG | AGTGCCACCAGCGATAAGAAAA | CAAGTAAGTGGTT[A,C]TATTCT | - |
| OMS00070 | 1 | 55279296 | CGTTCCTGCGGGACAGT | GTTTCTCTCACGTCCACAGATCT | AAATACGG[A,G]AATGCAG | + |
| OMS00003 | 1 | 59464348 | GTGCCACTGATGAGGATGAGATCA | GTAATAAAGCCCTTTTGTGAGGAAAAACTAAT | TACTGTCG[A,C]CATTTTA | + |
| Omy_gdh-271 | 1 | 63738413 | AGGTCAGTCTACTTACAGTATAAAGCAGT | GTCATGTCAACAGAGTAACATAATAAATCTGC | TCACCCTGAA[G,A]TGTAGAC | - |
| Omy_cyp17-153 | 1 | 64509889 | GCCCTCCAAGTTCCAAGTGAAAA | CAGGTCATTGATGAAACGTCAGAAC | ATACCTGAGT[G,A]TCATCG | + |
| OMGH1PROM1-SNP1 | 1 | 70466474 | TCAAACTGCATTTGATGGAAACAAACAT | AGGACAATTCTAAGTGACCTCAAACTG | TAGTGT[T,A]CACTGACTTCA | - |
| OMS00008 | 1 | 77384035 | CCCTTTAAGGAGGATTTTAAATATGTGAGATAGAA | GGATACAGCGTTTTGGAATGAAACT | TCAAATATCCATAAT[T,A]ATATC | + |
| Omy_stat3-273 | 1 | 79898098 | CAGACCTCCTCTATCTCCCTATGAG | ACCTCCTTTAAATTGTGCCCAAGAA | [C,T]CAGTTTG | - |
| Omy_G3PD_2-371 | 2 | 6083946 | GCAGGTAAGGTACACCATAGAGACA | CTCCCCCTGCCTTACCAAAC | AGACATGTG[G,T]ATTGGCA | - |
| Omy_98683-165 | 2 | 8555140 | GCCATTGCCAGAGAATTTGGTTAA | AACACACGCACCATCTTAAAGC | CCAGATACA[T,G]ATTTGT | + |
| OMS00156 | 2 | 12397197 | GAGCAGAACACATAGAGGAAAGACT | GTAATCACCCTCTTAGCCTGTATGG | TGTGTCCTGC[T,A]GTAACA | + |
| Omy_114587-480 | 2 | 16204563 | CAGATTACGTTATTACGTTTGGGAAATTTTTAAGT | GTGAAAGAGTGGGAAATATAATTATAAGGTCAGA | CCTGTCCA[A,C]AATTGT | - |
| OMS00138 | 2 | 22318448 | TCGGACCACATGAGCAGTTC | GTTCAACAGGTGCCCACAC | CTAACAATAACCA[A,C]AGACTG | - |
| Omy_NaKATPa3-50 | 2 | 23893157 | GTTGAGCGTGTTATGGGAAAAGAG | TTGCATCGGCTTTCTGAAAACC | TCTGTTTCC[T,G]TTCTTT | + |
| Omy_u09-53.469 | 2 | 48859883 | ACAGCCTGAGCGTTTGCA | GGAAACTGGGAGAGATCAAAGGA | TTGCAGCCCTT[A,G]TTGTG | + |
| Omy_metB-138 | 2 | 53792050 | TCTGTCCCTGACGCTATAAAAACG | GAAGTATTTCAGCTTAATTTCACTGTTGAGTT | TTCGCCAAAG[A,T]GAAAT | - |
| Omy_lpl-220 | 2 | 60022813 | TGACAATCACTGAGCAACTGAACTC | GTCCAGTCTTGCTTCAACTCATTCT | AGT[G,C]ACAGTCA | - |
| OMS00096 | 3 | 29109394 | CATGAGAATGGATCAGTCTCCACAA | GATGAAATCTGAATGTGTTGACACTACAG | AAAGAGGAAG[A,C]GTCTCG | + |
| Omy_112301-202 | 3 | 37590554 | GTAAACCCTGCCCACATAATTAGGT | CTGAGACACTGCTCCAAGGT | AATGCGAAG[A,C]CAAACT | + |
| Omy_u09-54-311 | 3 | 42920502 | GTGGCTCCCCAGGAACAAG | AAGTTTCATGTCACATTCCAGTTACCT | TGGTAATTATTCAACA[G,A]ATCAGT | + |
| Omy_RAD58835-15 | 3 | 53735338 | GTCTGCTAAGGTCCTGCAGG | GCCGACCATGAGAGACCTG | ATAGCT[G,T]CTGGGACCCA | + |
| Omy_u07-79-166 | 3 | 57523676 | CCCGCTATATTATTTGATCACCCTTGA | ATTTAAATCCATTTCTAAAAATAAGCAAACCTAACCA | CTTGGGAATA[C,A]CCCAGCC | - |
| Omy_117815-81 | 3 | 67894257 | CTGCTTTATGCACACCACATTGT | GCTCTTTCTGGAGAACAAGGTACTG | CTATACGGA[G,A]ACCAGC | - |
| Omy_118654-91 | 3 | 75458069 | CAGCGTAGACCGTTTCCTCATTAT | GCGCCGATGAGCAGCTT | CAGCTTGTC[T,C]TGCCGC | - |
| Omy_aromat-280 | 4 | 3391469 | CTCCATTGATTCATGCCGAACATT | GGAGAGGTCAAACATAGCCTGGTA | TCTTGC[A,G]AACTCC | - |
| Omy_105075-162 | 4 | 7635334 | GGAGAAGGACAAGGACATTGGTAAT | AAAGCAGACCACACCATACTTCTC | CTTTCTCTCCT[A,C]CTTTCC | + |
| Omy_myoD-178 | 4 | 10520793 | GGTCAAATATTTCATTTACGATTACACTTAGGC | TGGCAAAGCTGTCATTCCTTCTAAT | TTTTATGAGATAT[A,C]ATTTCC | - |
| Omy_128923-433 | 4 | 16436234 | CTATGTCCTTGGCAGAAGTCTACA | ACGTTTCTTTGGGCTGAGACTTATT | CATTTTCATTC[A,G]CTGTTTT | + |
| Omy_130524-160 | 4 | 28889024 | CGAAGGTAGCGATTGGTCGTT | TGTCTGTTCTGCTGTGTGCTT | ATGGCTT[G,C]ATCCTCA | - |
| OMS00079 | 4 | 35250473 | GTAACATTATGAATCTATCAGTTTCCCTAGCT | ACCTGCAACGTTAGAGCTGTTTATT | CTACTTTTCACAGT[A,G]ACACAG | + |
| Omy_117286-374 | 4 | 52310165 | TGATGTGTTGTTCCTCATGGCTTA | CTGTGCATTTATTCTTGTGATGCTAGG | TCCTCATCATAC[T,A]CTATGG | - |
| OMS00087 | 4 | 52511964 | GCAAATTTCACCCTTAACGTGGTTT | GATTTGATGTGTGTGTATTACCTCCTCTA | GTTAAA[A,G]CTGACAAAGTGT | + |
| OMS00111 | 4 | 63031581 | CATGCGGACCTGCATAGCT | GCTTAGCCATTGACAGAGCATATCA | AACCAGACT[A,G]CCATTC | + |
| OMS00116 | 5 | 11341754 | GCCTTTCTCCCATATCACATTCGA | AAACGCATCTTACACTGTGTTGTG | TTTACATTTTCAAT[A,T]TTCTG | + |
| Omy_110362-585 | 5 | 14307279 | GCAGCCAAGATGAACGAAAACTTC | CCGGCCTGGGTCTCAATG | CACCGCC[C,T]TGCCCGT | + |
| Omy_bcAKala-380rd | 5 | 53469295 | TTGCTCTCTTCTGGTTGCCTTA | CTTCAGGAGAAAGCGCTACTGT | CATAC[C,T]CATCCTATGTCAG | - |
| Omy_u09-61.043 | 5 | 58840810 | TAGTCACATCCATAGTAATACTTCC | TGTTCAGAAGCAGAAAACCAATCTCT | CTTGGTCC[T,A]TTTTCA | - |
| Omy_109525-403 | 5 | 84224892 | CCTCATTCTCATTGGTGAGTTGTCT | TGTAAGATCTGACCACATGAGTATAACCA | CCTACACCTCTTTT[T,C]TCCACA | - |
| Omy_105714-265 | 6 | 7594847 | CCACTCAGTGCAAGCATGGA | GCTTTCAATCCTTGGCTCCAATATC | TGTTGTTTGAG[G,A]TTCAG | - |

Table A2. Continued.

| **Locus** | **Chr** | **Position** | **Forward primer** | **Reverse primer** | **Probe** | **Orientation** |
| --- | --- | --- | --- | --- | --- | --- |
| Omy_107285-69 | 6 | 9809156 | GCCCTTGTGACAATGCACTGTTATA | AGGTCTAGACAGTGTGCCATTTG | ACGTTACTTTT[G,C]ACCTTGT | - |
| OMS00013 | 6 | 10325271 | GCCTTTGTTCTCCTTGGTGGTTA | AGAAAAGTGTGGACTGAGGTTGAG | CTTTTCCCT[T,C]GCTACTC | + |
| Omy_b9-164 | 6 | 17599731 | GCACAGAACACAGCCAATATTAACA | GCCTTGACTCTCCCTTCATGAC | CCTACAACTTGATCTA[T,-]CGTG | - |
| Omy_vamp5-303 | 6 | 33625138 | CTGCTTCCCAATTCAGTATCGTCTT | AGGCTGAAGCATTTCTGAGTATGAA | TGGCCGTAG[-,TAG]TTGGTCA | - |
| Omy_star-206 | 6 | 36624863 | CGTGTGCCAGCCCTTCT | GACCACTGAGATCATTGCTGTGA | TTTGGCAC[T,C]ATATCT | + |
| Omy_IL1b-163 | 6 | 42313129 | GGAACAACAGGATTAAGCCTACTCT | CCTAAAGGCCTAGGAAACTAAACTTCA | CTGAGGTCATA[A,C]AAATA | + |
| Omy_cd28-130 | 7 | 19036977 | CACAACTCCACAGAGACAGTGA | GAGGACAAAACTGACCGTATGGT | CTGTTC[A,G]TTCACCC | - |
| Omy_131460-646 | 7 | 36600038 | GTGAAAAGGAATGGAGGAGTACAGT | TGCTAGGACAGGAAGATCATTTGTG | AAAGCAGAATTT[G,A]TTACTG | + |
| Omy_pad-196 | 7 | 44470619 | CAAACAACCACAGTAGTCCTCCAAT | GCTTTTCACCCTTTTGTAAATTAAGCCAAA | AAGACAAAGGT[G,A]TAATACC | + |
| OMS00064 | 7 | 45227750 | GTGGATATGTAGTTCGATGGAACAGT | TTTACAACAATCTTCTTTTAATAAAAATATAGCCACTTAT | CAGGCAACATTTTAT[A,C]TAACTA | - |
| OMS00154 | 7 | 56234573 | GATGTTGGCTGGAGGTGTAGT | TGGGAACACTTTGCCTACCC | AGGGCTTC[T,A]GATTGA | - |
| Omy_sys1-188 | 7 | 56428776 | CTTAAATGGTGCTGGTTGCTGTATT | AGTGATATCTTAGTGGGTCGAGGAAA | AAACATGTAC[G,T]ACCTGTC | + |
| Omy_arp-630 | 7 | 57239751 | CTGCACAACTTGTTTCCTGCTATT | ACCAAGTGTCCCTGTAAGCC | CCGCTC[C,T]GTCTGCT | - |
| Omy_97077-73 | 7 | 61875465 | GTGTAAACAAAATGACTCTGGGATTCAG | AGAAGTGGCAATGGTGTGAAGTAT | TGGTGCAATAG[A,T]AATA | - |
| OMS00057 | 7 | 67908135 | GAGAAAGGGAGCATGAGACAGA | GTTGGGCTCCGGTACGAT | CTCCACAG[A,C]ACCTTG | + |
| Omy_105105-448 | 7 | 68303584 | CAATTTGCAAGCAGGGAAAGGTTAT | GTGATGGGCTGCAATTGCTT | AAGGAGAAT[G,A]CATAATC | + |
| OMS00132 | 8 | 9451350 | GTTTATGACTCCATTGCCGAAATGATT | ACGCGACCTGCAATTCATCAATA | AGCAGTCCTC[T,A]GTGTGG | - |
| Omy_hsp90BA-193 | 8 | 10667361 | GGAATCGATGACGACGAAGTGATC | TTCCTCCATGCGTGATGCA | CCTCCGC[G,A]CCTGC | - |
| OMS00153 | 8 | 16303316 | ACTTTGCACCATAGGCTTGACAT | TGATAAGGATGATCAAAAAGCTGAAGTATGTA | CAAAATGT[A,C]ATTTTCC | + |
| Omy_hsf2-146 | 8 | 17470364 | CCAACAATTGCAGCCTCATCTTAAT | GGAGCAGAAAAAGGATTGGACCTT | ATAATCTA[C,A][T,C]A | - |
| Omy_srp09-37 | 8 | 21426823 | TAGTTGTATTAACTCTTCTTTGAGTCTAGA | TCATTCCAGCTCCGTTCTCTTC | TTGTGCTATTGAC[G,A]CCACAG | + |
| OMS00151 | 8 | 23949230 | CTAACGTCTTCCCAATGATATTTCACAAGATA | ACCGTGGAAATACAATTTTTTATGCCAAT | ATGACCT[T,C]GATAATC | + |
| OMS00179 | 8 | 25539930 | GTCATAACAAAATCAGGGCTTTCCAA | TGGGAGATTTGGGCTGCTTTAAA | CCTCTTCTCTT[T,G]TCTCAT | + |
| Omy_120255-332 | 8 | 29102820 | GCTAGCTAACATTGAAGGGTGGAAT | GGCTACAGGGACTTTACAATGGG | ACTATGCCA[T,A]GAAGTTA | + |
| Omy_104519-624 | 8 | 42764123 | CGTGTGAGTTTGCGGTAAAGAC | TGACGAGTCCGTCTTATCATCCT | AGCAGGATAC[A,G]TCCGACT | + |
| Omy_108007-193 | 8 | 43671991 | GTGAATACCACCCAGGCTTGT | GTCCCTTCCCCAGTTTCACTTAATT | TTTTCTCCC[T,C]ACTTAAC | - |
| Omy_nkef-241 | 8 | 69427037 | AGTGTCATTGATGTCGGCCTATTTT | AAACGAATGTCCACCTCAGATGTT | CTTCTGTAT[C,A]ATTTTTG | + |
| Omy_97865-196 | 8 | 82513344 | TCCAGACTTCTGGTTTGTTCCATT | CCAGCCCCTATATTCACAATTAAGTGT | ATTAATT[A,G]ACAAGCT | - |
| Omy_rbm4b-203 | 9 | 12485117 | CTGAAATTTGATGAATGGAAGCTGCA | CGTATTCAAGTCGATATACAGTCACGAT | ACGTTATTATG[A,-]AAAAGGATGT | + |
| Omy_112820-82 | 9 | 21861264 | CCTTTCCTTTTGCATTTCCTCTACTTATTTATTT | AAATGAACTCACGTTGACCTCTGA | CGCCGC[C,T]AAGTTA | - |
| OMS00103 | 9 | 38335692 | GAGATCACTGTAGGATTGGCTGTTT | CCTCAGAGCAGCTCACAATGGCATC | CCACAGTAATT[T,A]TTTTTT | + |
| OMS00056 | 9 | 43771325 | TCAGGAAGTAAACTGAAAATTCCAATGTATGA | CCCCAACCATGCTTGTTATTGAAC | CTTGACC[A,G]AATAGCA | + |
| OMS00175 | 9 | 50381707 | TTGCGATATGGGACTGTATACATTTATTCC | ACTACCTCCAGTTAAAATAGTGTGGGAAA | ATCACTAGTTCA[A,G]ATACAA | - |
| Omy_tlr5-205 | 9 | 60510483 | GAGCGTATCTGGTATGGTAACAACA | CTCCAGCAGCTTTAGAGAGTTTACA | CAGTAATATTTC[A,T]GTGCCCG | - |
| Omy_114315-438 | 9 | 64564508 | CCTCACCGATCTAGTCAACTTCATC | AGGAGGCTGAGGGAGATTCTAG | TTATGGGCTTA[A,C]GGGTC | - |
| Omy_gluR-79 | 10 | 7508221 | GACTGTCTATAGCTATTCTTCTCAAACTGT | AGAAACTACCATTGTGATTAACAGATAGAAAATACAT | CAAGTATTTTGC[G,A]TAGGAAT | + |
| Omy_109894-185 | 10 | 10852282 | GGGAGGAATTGGAATGACAGATTAAC | CGGTGTCATTATGGTTGTCATTGTG | CTCCCTG[A,G]TCCCCC | - |
| M09AAE.082 | 10 | 32872704 | CTATGTGCAGTGCCCTTCTCA | GGCTTACAAGTATGCATGACTAGCT | AGGTTGTTTTACA[A,C]ATTTAA | + |
| OMS00095 | 10 | 38704654 | CTCCAATGGCTGTCAACAATTAAATATAAGAC | GTGTGCTGGTCTCTTCTTTTATTCTCA | AGGCAACTATATAT[T,A]TTTTT | + |
| Omy_RAD26080-69 | 10 | 40486618 | TGTGGGACAGCACATACTCC | CCAGGACACCAGTGGAGAAG | ATTAGTA[G,A]CATCATCGAG | + |
| Omy_ftzf1-217 | 10 | 44060919 | ACAGGGATGGGCAACTTTGTT | GGATGACCCACGTGACACT | TGACGAGTTC[T,A]GATTT | - |
| Omy_p53-262 | 10 | 46190822 | CCCCAACATCCAGTATACAGTTTCA | CCCAAATTGGCAATTTTAATAGGATTCAGA | AAGTAGTATGG[A,T]GCTCTAT | - |

Table A2. Continued.

| **Locus** | **Chr** | **Position** | **Forward primer** | **Reverse primer** | **Probe** | **Orientation** |
| --- | --- | --- | --- | --- | --- | --- |
| Omy_tlr3-377 | 10 | 56447078 | GTCGCTCCGGGTGCTT | GGCCCAAACACTTCCTTCCT | CGTGATTAG[G,A]TTCTTC | + |
| OMS00106 | 10 | 59855697 | CGTGTAGCATTCTTGAGGAAGCTT | TTTCCAACAGATGCCAGAATCCT | TGATGG[A,C]AACTTTC | + |
| OMS00030 | 10 | 60401405 | CCTCGTGACTACAGAGCTATACAAC | GATCTGATCGGTCGGGAGAGA | ATGAGGGTCCCT[A,C]TACAGG | + |
| Omy_UT16_2-173 | 10 | 61496264 | ATTGACTCATTATCACCTTAGTTGTAGCTTCA | GCAGCTACTTGCTGTATCACATGTTTGT | ACAGTCAA[C,T]AAGGGACTTAA | + |
| Omy_RAD36848-7 | 10 | 63650747 | CGAGGACGTTCATAGGGAGC | TCGATAAGTCCACCAGCTGG | TGCAGG[G,A]ACACCACCCT | + |
| Omy_g12-82 | 11 | 14764807 | GATCAATTCGATCGCTCATGAAACTT | CTTCTCTCGTTCTCATTGTGTCTCA | AAACTCTC[A,G]GGATTAG | + |
| Omy_BAMBI4.238 | 11 | 24411085 | CATGATGAGGAGGACCAAGATGAG | AGGTGTGGTTCAGGGCAG | ACCGC[A,G]ATCACCG | - |
| Omy_MYC_2 | 11 | 33841260 | CGGTTGCAGAACTCTCATGTTTG | CACGCCATGTCTTAACTTGCATTA | CATAGACTTTTTG[A,G]CCTTAT | + |
| Omy_BAC-F5.284 | 11 | 42789302 | CCTCATTTACTGTAGGACCATGCA | ACAACGCCAACAACTTTCTCTTG | CAGTAGG[G,A]CGGCAAG | + |
| Omy_cox1-221 | 11 | 47052310 | CACTGAACTGTAAGCCATTGTGATT | GCAACATGGGAATGATTCATAAATGCA | CGGTAAGACCATT[A,T]AAA | - |
| OMS00120 | 11 | 51013177 | GGCAGAAGAGGAGAGAGATATGATTG | CCTCAAATACCTCTGACATTGAAGGTT | CGCCCAC[T,C]AAAAC | + |
| Omy_102867-443 | 11 | 55934332 | CATTTGTTTAATTTGATTTGGCACAACTTCA | CCCTAGTTCTGTAACACAAGACGTAA | TGGGTACAT[A,C]ATTTTT | + |
| Omy_oxct-85 | 11 | 68405894 | CGTCACTGAAACATTACTGTAACATCCA | CATCATCACGCTGTTGGTTTCTTAA | CATCGCT[T,A]ATTTATGC | - |
| Omy_117540-259 | 12 | 5079371 | GGCAGGTTAACACAGTCATCTACTATAAA | CAGCATGTTGCTTTAATCCTTCACA | TGTCACTTCAA[A,C]GTTTG | + |
| Omy_110201-359 | 12 | 28727952 | GGTAAGGCCTGTCTGACTATTTTGA | AGAGGTCAATGGATGCCAGTTT | TTGGCTATTGAAATT[A,C]TACATT | - |
| OMS00074 | 12 | 31104727 | CCTGTTTATTCATCTAAACCAGTTCTTTAAAAT | AACTTAATTTAGCAAACAAATGTCTGAACAGAA | AAACAAAACA[A,C]ATGTTCC | - |
| Omy_hsc715-80 | 12 | 40136309 | CCGGTCTACCCTATAGCTGTTG | AGTCAGTCAATTAGTGGTTTGAAATACTATCA | AACTGTATTTG[G,T]GAAAAT | - |
| OMS00077 | 12 | 48861950 | AATACCATCTTGAGCTCATTAGTAATTATTCAA | CCAGACTTTACACACTCTTGACTGA | CCGGTG[G,C]TGAAGTT | - |
| Omy_gh-475 | 12 | 62308122 | AAGTTACCAGAATTTTGCAAACTCAACT | CCATATTTTGAGGTGTAGCTTTACCCT | CTGAAACTCATG[G,A]TATACA | - |
| OMS00149 | 12 | 65894619 | GGCATCATTGTTCTTGCTCTGTTTA | CCTGGGAGGGTTTATATCGGAGTAT | GCTAAA[T,G]GCACAG |  |
| Omy_hsp70aPro-329 | 12 | 66828836 | TGCGTATTATTGTTTTTCAAGGACTTTCAAA | TGAATATTTTCAAATACATGCCAATTCTTTCCAA | CATTCCAATAT[T,C]CAACTAT | + |
| OMS00112 | 12 | 68382081 | TGGCAGCAAAAGGGATGCA | TCCTGAGCAACCAGTCAACATT | CGGTTTCAAGT[T,A]TACTTGT | + |
| Omy_111666-301 | 12 | 70990844 | GGGTGAAAAGAGTGGGACATTTACA | GTCAATTTCAAGGCACCAGACAAT | AGTATAACACAGT[A,T]AGACAAT | - |
| Omy_118175-396 | 13 | 20282478 | AGGCTTCACACACACATGCA | GACGCGCAACCTCTAGATTATACTT | CTCTTGCAGACAT[A,T]CCCGTA | + |
| Omy_129870-756 | 13 | 22915161 | TCGTTATTTTGCCTCGCGGTA | TCCCATGAAGATGTATACATGTTTTGTGA | CAGGTATTTC[G,A]TGAAATG | + |
| Omy_113490-159 | 13 | 26494831 | CATAGTACATTTACAGATAATGTTTTAAAGTGCATGT | CGAGATACCAAAATGCCACAGTTACAT | CATCTGTTTT[G,A]GTTTAGC | - |
| Omy_nach-200 | 13 | 30001796 | CTCATGAAAAACGGGAGAGCAAAG | CAGCGGCTCTTCAGTAGTCT | CTGACAGAG[T,A]CACAAC | + |
| OMS00180 | 13 | 32462775 | GCGCCGAATGGCATTAGG | CACATTGCTGTCGTTTAGTTTGACT | CTAAAAGTGC[A,C]TTAAGCC | - |
| Omy_110064-419 | 13 | 36272850 | GTGCAAGGGACCTAGCTAATCC | TCTGAACTGACACTGAAGAACAAAGAA | ACGTTAGCTTTT[A,C]ATTTC | + |
| Omy_g1-103 | 13 | 39287232 | AGTCGTGACAATGAGAAACAGTGTT | CTCAGCAAAAAAGAAACGTCCCTTT | CTTTTACA[A,G]TGAAGATC | + |
| Omy_IL6-320 | 14 | 7102407 | CGACTGATCTCCTGCAGACATG | CTTGTTCCTCGTTGTCTTCCTTCTA | ATAGGAGAGA[G,A]GACAACA | + |
| OMS00089 | 14 | 13150534 | GCACCATTTGAATAAAAAATCTGCTTTGT | GCAACCCAATTCAATATTAAGCACATGAT | AATCCCAAA[T,C]AAGAAC | - |
| Omy_116733-349 | 14 | 18498042 | GAAATGGACATGCCTACAAATTGCT | GATGTGATCAGTTTAGGCAAGGC | AGAGAATCTGATA[G,A]TATTTC | + |
| Omy_ntl-27 | 14 | 22071034 | GGTGTGTTACTGTAGTTGTGTCCTT | TGTGTAGCTAGTGATCCTGATTGTCT | CAGACAAGAGTAC[C,T]CCAAGAC | - |
| Omy_txnip-343 | 14 | 24435825 | CCTTCAAACTAACGCATCATAGACATG | GGTCACTTGGCTAATCCCCTTAT | AACTGAAG[A,G]GATCTG | + |
| Omy_UBA3b | 14 | 28552649 | GCCACTCAATGCATGTGTTTTCTAG | CAGCTAGCTTAAGTGGGATGCAA | AGATAACGC[T,A]AACTATT | - |
| Omy_mcsf-268 | 14 | 48758470 | CCAGCATTCGTTCCCATTTCC | CTTTTAATGTAGATTATATTCTTCTGTAGCCACTATGG | AAATAA[T,C]AGATAAACCCT | + |
| Omy_101554-306 | 14 | 53660117 | GCCTGTATTTCTCCTGTATGTGCAT | TCAACTTTTGCAAACTTTTTTATTCTTTGTCATTT | TGCTTCTCAC[A,G]TTTTTA | - |
| Omy_RAD47444-53 | 14 | 56051917 | GTCGTCTGGAGGAGCTGAAG | GGGTGACGTTTTCCTTCAGC | GGCGAG[C,T]TTGGCCCAAA | - |
| OMS00072 | 14 | 59971780 | GTGGGAGAGCTCGTCTATGG | ACAACAGGTCATTGGATGTGATCAG | AAGGTCCATG[T,C]ATCTC | - |
| Omy_redd1-410 | 14 | 69044121 | GTACTCCCACTAACATACAGTAGACTCA | GGCACCATTGTGTTTTAGGATGTAG | AATATCCTGCAAG[G,A]AAT | - |
| Omy_nxt2-273 | 14 | 71969784 | CTTTAGAAAAGCCAAGGTATATTTTAACATACTTCT | CTGCTGCCCTCTAATGGTAAGATAG | AAGGCA[C,T] | - |

Table A2. Continued.

| **Locus** | **Chr** | **Position** | **Forward primer** | **Reverse primer** | **Probe** | **Orientation** |
| --- | --- | --- | --- | --- | --- | --- |
| Omy_110689-148 | 14 | 72350872 | GTGTGTGGCAGAGAACTAACTGAT | GGTTAAGACATTAACATAACACTGGACTCT | ATGAACACAT[T,G]ATTTATC | + |
| Omy_Ogo4-212 | 15 | 12595806 | TGAAAGGTTTTATGCAGGTTATTTTCT | GTGTGTGTTAAATAAGCATTTGATGA | ATTTGATGAG[A,G]CATCTT | - |
| Omy_hus1-52 | 15 | 13042222 | CTTGCCGGAGGGTAGCT | CCACAACTTCTCAAATGAATGGAATGT | CCCATCCCT[C,T]CTCCTGG | + |
| Omy_111084-526 | 15 | 17300850 | CACCACACCAAGCAACTATTTCATT | ACCCAACTACTGTCCCATTTTTCAT | CAGTGAAAT[T,G]TATTTTT | - |
| Omy_111383-51 | 15 | 21239768 | CACGCGCAATCTCTCGTTTTAC | TCTTTAGGCAACAAGCGTGTCA | AGCAAG[C,T]GCACTAGGT | - |
| Omy_carban1-264 | 15 | 21454064 | GCAAAGCCTCATCTTCAATCATTTGT | GCAAAACACAAGTCAGGAATCACTTA | ATTAATATTGCTAATAACAC[C,T]AAG | + |
| Omy_96222-125 | 15 | 24041111 | GTAAGGAACTAATTGGCGCAACATT | CAGTTTGTCTAACACCCAGGCATAT | CAACTGT[A,G]GCTAATT | - |
| Omy_RAD52812-28 | 15 | 28623729 | AGGAGTCCTGTCCCATGTCA | GCTTAAGGCTGTGGTATGTGG | CAACCT[C,G]TATTCCACAT | - |
| OMS00061 | 15 | 31231975 | AAGTGGAGGCTGACCTGTTG | GCTGATGGCACCTGACAGTTAATT | TGCCATTT[A,G]CAGACTT | - |
| Omy_nips-299 | 15 | 39788784 | GACAGGATAGGAACGGTTTCTCAAT | ATCAGAAGTTTAATTCAATATGTACACGATCCT | CTGGATTTCAC[A,-]GTAATAC | - |
| Omy_LDHB-1_i2 | 15 | 44094440 | ACGCACACTTATCCTTGACAATGTT | ACTGTGACAACAAATTCGGTGACA | TGGGCA[G,A]TCATTCA | - |
| OMS00143 | 15 | 59233015 | GGAGGCACGCCCCAAA | TTTGTTAAAATAGAGCCCTTAGTGGGTTT | CCTGATCCAGA[A,G]TCTAGA | - |
| Omy_97954-618 | 16 | 11433435 | GCTCTGCTTCCTCGGCAAATA | CACAATTGGTTTTTGCACAAAAGTAAAGTATT | CAACGCTTACC[G,A]GTGTGT | - |
| Omy_117370-400 | 16 | 14853962 | TGCAAACACAGAGGAAAGGGATTT | GGCTTATTTGTTCCGTACTTGCATT | AACTCCAA[T,C]GAATTAA | + |
| Omy_RAD88122-32 | 16 | 16334074 | TCAGTGGATGGAGTGTCCCT | GGTCTTTGGCCTTGTTGCTG | GCTGTGGA[G,A]ATCATCCG | + |
| OMS00041 | 16 | 18279174 | GATTCTGTTCCATCCTCTTTCTGTCA | AAACATAAAAAAGGGCATGAAGGTGTC | CACTCTATGC[C,G]TGCCCT | - |
| OMS00119 | 16 | 27506018 | AGCGGCAGTTGTGTTAATGAGA | CTTCCTAAAGCCTGACAGTCTGT | CACACAGC[T,A]GCCTGT | + |
| Omy_anp-17 | 16 | 30728525 | GGTAATGCCACATGCGGTAAATT | GGCGAAATCTGAAAATGTGCTGTTA | CTCATTGGTATA[G,T]TAACC | - |
| OMS00018 | 16 | 46432462 | AGAGTACATGTGTGGCTGCAA | GTCATAAATCAACACAATTATCTTCTTCACAGAA | CCACATAATT[A,C]ATAATTC | + |
| OMS00134 | 16 | 47743637 | GAAACTGAAATGATCCCATCGTGTT | GCTAGCATAACAGCATTGCCATAT | TAGCTGCAG[T,C]ATATTA | - |
| OMS00006 | 16 | 63247944 | TCCACGTAGGACATAGTTTGAGCTA | TGTGGTGTCATGTTTGCCCTAC | CTTACAAAT[A,G]CAAAATT | - |
| Omy_103705-558 | 17 | 7065986 | CTCCAATCGCAAATACCCAGACT | CGCAGGAGACGGATGCC | ACTTACCCAG[A,G]GTGAGAG | - |
| OMS00128 | 17 | 14488587 | ATGAAAGAACTCCCAGACACGTATTTT | ACATTTTAACACAGTAACACTAATACACACCA | ACTCTCAGAATT[A,C]ATTATG | + |
| Omy_101832-195 | 17 | 17015658 | TGGCTCTGGACCTGTTGAGA | CGTCACAGCTATTTTAGGCGTAGT | TAGTCTTTCAGAG[T,G]AGTATG | - |
| Omy_RAD45104-18 | 17 | 20693754 | TGGTGCTTCAGTGCTGTCAA | AGAGTGAAAACTGTGTGCGG | CAAGAC[A,G]CCGCACACAG | + |
| Omy_101993-189 | 17 | 21491290 | ACAAAACACAGTGGAATTACAATTAACGTT | GGAAGTTAAATTTCGCTTCGTCAGAA | TGATTTGCAGC[T,A]TGTCAA | - |
| Omy_114976-223 | 17 | 41462973 | GACAAACAGCACTTCATTGCAGTAA | GTTGCTCCAGCACCAGGT | CCGATGG[A,C]ACAATC | - |
| Omy_u09-56.119 | 17 | 41690956 | CCAAGGTGGACCCACCAG | GCTGAGTTTATAGGTCAGTCATTATACATATTGA | TGAGCTGAA[A,G]CAGAGCA | - |
| Omy_ca050-64 | 17 | 48651793 | GTCATACAGAACTGTTTTGTTGTGTCAA | ACCTTGAATTGGTTCCTAATGCTATTGT | CAGTTTGAAGA[A,C]TATACTC | + |
| Omy_RAD58213-70 | 17 | 58266227 | CCTGATGGGTGCTCTTCTCTC | AAACAGCATCATTATCCATAGTGTT | TTTTTT[T,A][A,T]AAATATACT | - |
| Omy_U11_2b-154 | 17 | 59466696 | GGGAAGCAGAAAAACTGGAAGTT | CCCTCTGTGGGCTTGATATTCA | TGATACTTTTCAG[A,G]TTGTAAC | - |
| Omy_sast-264 | 18 | 28252083 | GAAGTAGGGTTTGTTGACCATGTGA | TGGATTCCATTTTAGGCTGTAATACATCTT | CTAGCCAATG[C,T]GTCTAA | + |
| Omy_RAD43612-42 | 18 | 29118777 | GTGGAGAGGGATTTTGGGGG | TGACAGGACAAACACAAGCCA | AAATGTG[T,C]ATTTGTGTA | + |
| Omy_128996-481 | 18 | 30802101 | CTCATCCACACTGTACAGTACAAGT | CATGCCTTCGTCTCATCAATAACAC | CAAACC[T,G]CAACCAC | + |
| OMS00121 | 18 | 34232991 | GGAAGGAGGTCCAGTGTGAGT | AAAATATGCAACACCACTAAAACTGGAAAA | CAGCGTG[A,G]TAAATT | - |
| OMS00127 | 18 | 36268423 | CACCTTTCTCTCTCTCTCCATCTCA | AGTGTGCTACACAACCTTAAAAAATATATATCTATT | ACACACCC[A,C]AATGTA | + |
| OMS00118 | 18 | 42212299 | GCTTATTTAGAGTGCATGCCAGATG | TGGAACCAATGGGACAGTCCTA | GCGGGG[T,G]GTGCACATT | - |
| Omy_RAD7210-8 | 18 | 42558654 | ACACCACACTCCACAAAGCA | GCGCCTTGGTCTCCTTCATA | TGCAGGA[C,A]TTGCTTTGT | - |
| M09AAD.076 | 18 | 53717512 | ACTGTTACCACTCTCTCATCAACCT | GGGTCCAGGAGGTTTTTAAACAACAT | CCAACC[A,G]CTGGTGAA | - |
| Omy_BAMBI2.312 | 19 | 6114061 | CGAGCTCATGTCCGAAACTCAT | TTTGACAGCCTCAACTTCTAGGG | CCGAAAGTT[C,A]AACTTT | - |
| Omy_Il-1b_.028 | 19 | 10329530 | ACTGTCTGGCTAGAGCACATTG | ATCTTCTACCACCGCACTGTTTTAA | TGAGGCA[A,G]CTTTTGT | + |
| OMS00092 | 19 | 12518551 | TCTCCAGGTGTATCTTGAGAAGGT | AGGGTTCACACAGGGAAGATATCAT | AGCTGAGAA[T,G]AGGTTC | - |

Table A2. Continued.

| **Locus** | **Chr** | **Position** | **Forward primer** | **Reverse primer** | **Probe** | **Orientation** |
| --- | --- | --- | --- | --- | --- | --- |
| OMS00017 | 19 | 19111591 | ATTAAGTTCATACAAAAGTTCATCATAAATATTTTCCTTT | GGAGAACAAAGGGAAAGAGAAGACA | CCTCGG[T,C]GCTGTAG | - |
| OMS00105 | 19 | 20264968 | ACATTTGAAGTCAGTATGGGTGTTGAG | GAACCTCACCACAGTACTAAATGCA | CTGCTATTCA[A,C]ATTGCT | + |
| OMS00133 | 19 | 23702093 | GACCACTTCACTCATTCCTCCTTTT | TCCGGTTTACACACTTCATGCA | CGCCTCCATCT[T,C]TGTGGT | + |
| Omy_rapd-167 | 19 | 27362424 | CCCAACATGCTCTATTGCAGCTA | AGTTGCATAAGATGAATCAATAAATTAAAAACACAGAT | AAACAATCCC[C,A]CCCAAA | - |
| Omy_128693-455 | 19 | 32890059 | GCCTGCAGGAGAAGGTAGAGTTA | GAAATGGAATGGACCCCAATCCT | CTCA[A,G]CTGATACCC | + |
| M09AAC.055 | 19 | 53855167 | GTCTCCGACGTGTGGCT | TGGAACGAACCTGAGAACATAAGG | ACCTCCAC[G,A]CTGTCC | + |
| OMY1011SNP | 19 | 54446225 | AGGCTGGTTTGGGATTCACTG | CGCCAAACACTAACTCTCTGTCT | CTTTACCTC[G,T]AAGACAAT | + |
| Omy_RAD29700-18 | 20 | 1673196 | AATGGAATTGGCCCCAACCC | TCTCCATTGTGTGTAATCATGGT | ACAATT[C,A]AAATGATTTA | - |
| OMS00039 | 20 | 4800495 | GTCAGTACTGTGTGTGTCTGTGT | CCATCTACATTGTCAGCAGTGTGA | GT[A,G]CGTGTCTCTGACC | + |
| OMS00114 | 21 | 11035495 | GGATGATGCTGTGAGTCGAGAAG | ACCTTCGCCACCCATGTTTTATT | AAACGTTTCAC[A,C]TGCACC | + |
| Omy_99300-202 | 21 | 14697073 | CAGTTTGACCCGATGGTGTGA | GATTATGGCGTGGCCTTTTGG | TCAGGCATG[A,T]GAGAAA | + |
| Omy_cin-172 | 21 | 21602618 | CGCATGGGACAGGTGTGT | GAGAAAGCCTGTAGAACCATGTCT | CGCTCACC[G,A]TGGTTAC | - |
| Omy_vatf-406 | 21 | 23806220 | TTGCTTCATTTTGTCATAACCTTGGG | TGCATGCTCTGACAAATGTTACACT | ATGACT[A,G]TCCACA | - |
| Omy_LDHB-2_e5 | 21 | 24129907 | TGCTAGGTGAGTCAGAGGTACATATT | GACTGGAAGGCCACCCATAAG | CCTGTC[A,G]AC | - |
| Omy_zg57-91 | 21 | 32766740 | CACTCATACACTCACTCACAAAGGA | AGCAGATAAGCCTTGTGAGTGAATCTT | CACAGACT[G,T]CACAGCC | + |
| Omy_b1-266 | 21 | 41255773 | TCATGTGAACTTTAATTGACTAGGAAGTCG | GATATGAAAATATCTGAAGAGTTATATTTGGGAAATTGAC | TCTATAAACAA[C,A]ATTTTTC | - |
| Omy_1004 | 21 | 41944218 | GAGAATCGGAGCTAATCTTAGTTATTGTGA | CACTTTATTGAGCTACATGGCAAATCTG | ATGTGATG[T,A]TTTTTGC | - |
| Omy_102505-102 | 22 | 7759328 | CTGCAAACTGACATGGTAGCAAAA | TGCTTGCTTTTTAAAAACAATCTCCCA | CAGGATG[T,C]TTTTGC | - |
| OMS00173 | 22 | 9105891 | TGGAAGTAGCTACTTAACAGGAAATGG | AACACGTGTGCTTGTTTTGTCAA | ATTAGCTTGTGT[A,G]TGAACT | - |
| Omy_Ots249-227 | 22 | 18917430 | CTATCTATCTATCTATCTATCTATCTATCTATCTATCTACTTACTGAGA | CCCCTAGATTAAACCTGTCCAGTCT | CCTCTGA[G,A]AACTAC | + |
| OMS00058 | 22 | 19922139 | GTGACATTTGGAGCCACTGC | GCTAGGAGACAGAGGGTGAAAG | CACTTTG[T,C]ACCCCTC | + |
| Omy_IL17-185 | 22 | 27226063 | CCACCACACTCTGCAGCTT | TTGACGGGAATCCGAGACTTC | AAGAATCTCAC[C,T]TGCCCAT | - |
| Omy_107806-34 | 23 | 10095418 | TCTTTGTCCATGCACATTGATATT | AGCACATTTAGTTAGCAGTGATGGA | ATTGGATGTCA[G,A]TGTCATT | - |
| Omy_RAD48799-69 | 23 | 21581080 | GCTGAGCCACCTACACACAG | GTCTAACACTCGCAGCAGGT | CATCCT[A,G]GAATAGAAGT | - |
| Omy_187760-385 | 23 | 23659737 | CGGCTATTCTCGCGTAAAAGCT | AAATGCAACCAGAAACGGAATGTC | CTTATCCAAAAT[T,A]ATTGTGC | + |
| OMS00024 | 23 | 31418339 | CACATACAACCATCACCCTTCCTAA | AGCATTGAGCGAAATTACCAAGAGT | AA[A,C]CCCAATTTTAC | + |
| OMS00048 | 23 | 37125668 | GGAAGAGCTGGAGAACAACGT | TGCAGTTGACAGAGGCTTTCTTT | AGCTAAACTC[A,G]GCAAAA | - |
| Omy_e1-147 | 23 | 38313338 | GCACTGACTGTTACCAGGAAAGAG | GTACTGCAGTGTTGAGGCTATATCA | CCATCCTGAAT[C,A]TGATTAA | - |
| Omy_109243-222 | 24 | 7009687 | ATGTGCACCTCTTAAATTGTAAGTAAAATGT | ACCCTATATTCAGTGGCAAGATTGC | TTCATTAAAT[T,G]GACTTTTT | + |
| OMS00101 | 24 | 10475451 | GCGTGTCGTGGGTCAGTTAAATA | GTGCAATCCAACCTATTAGTAGATATGCT | CTAGTAGCCTTA[T,C]AGAAAG | + |
| Omy_inos-97 | 24 | 24547687 | GATGGACAGGGTCCTCTTCAC | CCTGTAGATAAAACATGGTACCAGGTC | CCTTTCTTGAT[G,T]GTATCC | + |
| OMS00052 | 24 | 28174967 | TGCGTTTTTCATCCCAATCATTCAC | GGCATCAGGCTCTTCTTCCT | CCTTTTG[A,C]GAATAAT | - |
| Omy_107336-170 | 25 | 30017455 | GCCCTCTCACTCATGACATCAAC | GCTCCAGCCACTCGCA | ACTCCTG[G,C]GTGCAGAA | - |
| OMS00174 | 25 | 47325393 | TGACTAACTATGCAGCCTGAAAGG | GGGATACTCTTGTAATAAACTGTTGGTTAGTA | CAAGAACAGGA[T,G]AAATGT | - |
| OMS00071 | 25 | 48248474 | CCGGAGTGACCTCACATTTGG | GCATCGTACAGTTCACCTACCT | TTGTTTGAGC[T,C]TTTTCT | + |
| Omy_ppie-232 | 25 | 54990925 | CTGTTTTAGATTAGAATGTTTTTGGTCAGGT | CTGAACATAGGCTTTCATTTCAGACAT | AAATAGC[G,A]GAGAAAAT | + |
| OMS00002 | 25 | 82366395 | TTTGATTTGATTTGTATCTGCTTCTT | CCAACATGCCTCACACAAAA | TGTTT[T,G]GCAGCGCT | + |
| Omy_cd59-206 | 26 | 8028322 | CGATTGGCCCAGATGTTTCCAT | GCTCCGTTGCATAGGTGACT | CAACAATC[G,A]AAGGTAAAT | - |
| Omy_ada10-71 | 26 | 18315420 | TCTTTGAGCGACAAAGTCCTTGT | ACCCACACATGAACGCAAAAG | CTTCCTGC[G,A]TCCAA | + |
| Omy_aspAT-123 | 26 | 19289582 | GCCCATTTCACTGATGCTGTGA | AGGAGACCACTCCAAAGAGAACT | TTCCT[A,G]GGCAGTCAG | - |
| OMS00014 | 27 | 6297526 | CTTACACACAAGGGCTTCATTCTG | GATGTCTCTGGGTGGTTGTCA | TTGATGAATT[A,G]AACTTC | + |
| OMS00015 | 27 | 23730945 | TCAGACCCTATTTTTGGCACAAGT | GTCTAACTGATCCCACTTCTGCAT | CAAGTCACACTT[T,A]TAATGAA | - |

Table A2. Continued.

| **Locus** | **Chr** | **Position** | **Forward primer** | **Reverse primer** | **Probe** | **Orientation** |
| --- | --- | --- | --- | --- | --- | --- |
| Omy_hsp47-86 | 27 | 27944494 | CACATTAAGCACTCCCAGGGA | TTGCAAAGGCCAAACAGCATT | CAGGAGTGTA[A,T]ATGTTT | - |
| OMS00090 | 28 | 4245997 | AGGGCACAACACCACTCTAAATT | TCGAAAAGCAACATCTGTCTCAGT | AACCAC[A,G]CAAGATT | - |
| OMS00129 | 28 | 22531125 | GGAGATGATGAAATAAAAATTGAGGAAAAGATGA | TGTCTGGTGAATTATCGCAAATAACCA | TTGAACAACAA[G,C]AAAAA | + |
| Omy_97660-230 | 28 | 35742946 | TCAGTTATGTGTAATCTCATTACCTCTCCAA | AACAGAAAAGGTCTCAATGTATTTTTTGCA | ACGTAACTTGTA[G,C]CGTTTT | - |
| Omy_impa1-55 | 28 | 36390509 | CGCTGAGAGGATTGTCAA | TTTTCTTTGTTCAGTCTTCTGTCTCTG | CGAGATGATGC[G,A]TCTACA | - |
| Omy_BAC-B4-324 | 29 | 14528635 | CGTACTTTTCTTTTACAAAATTAAGTGGAGGAT | GCCTAATATTGGCCTAATGTCCTTCA | CATTG[C,A]CAAATACG | - |
| Omy_OmyP9-180 | 29 | 15673414 | CTGGATGTGTAGTATCGGTGGAAAA | CACTGGGCACCTCTGATCTC | CTGTAGTAGTCC[C,G]CATTGT | + |
| OMS00164 | 29 | 17249654 | CAGAGGAGAGGAGAGCAAAATACTT | ACAACCTACTCATTGAAACTCATTGGA | CAGATTCAATT[A,C]AATTTA | + |
| Omy_crb-106 | 29 | 30701374 | GCTCAAAAAGATTCTGCCAAATTCACA | ATTACAATGAAAGTACTTGAGTGTTTATGCAAA | TTGCAATG[C,A]GTCTTT | + |
| Omy_sSOD-1 | 29 | 39802221 | GCCGGACCCCACTTCAA | CAGACTAACCGAACAGCATCAGTGG | CCACAAC[A,C]AGACCC | + |

Table A3. Environmental data collected for all locations throughout the Columbia River.

| **Population** | **Migration Distance** | **Eleva-tion** | **Water Temp** | **Heat Load Index** | **Annual Mean Temp** | **Mean Diurnal Range** | **Isother-mality** | **Temp Season-ality** | **Min Temp Warmest Month** | **Min Temp Coldest Month** | **Temp Annual Range** | **Mean Temp Wettest Quarter** | **Mean Temp Driest Quarter** | **Mean Temp Warmest Quarter** | **Mean Temp Coldest Quarter** | **Annual Precip** | **Precip Wettest Month** | **Precip Driest Month** | **Precip Season-ality** | **Precip Wettest Quarter** | **Precip Driest Quarter** | **Precip Warmest Quarter** | **Precip Coldest Quarter** |
| --- | --- | --- | --- | --- | --- | --- | --- | --- | --- | --- | --- | --- | --- | --- | --- | --- | --- | --- | --- | --- | --- | --- | --- |
| Abernathy FTC | 88.21 | 15 | 14.74 | 0.850 | 95 | 104 | 43 | 4798 | 236 | -3 | 239 | 40 | 156 | 158 | 37 | 1775 | 279 | 29 | 60 | 823 | 136 | 148 | 758 |
| Agency Creek | 737.13 | 53 | 15.53 | 0.831 | 73 | 126 | 38 | 6988 | 262 | -63 | 325 | -10 | 161 | 164 | -14 | 570 | 102 | 10 | 67 | 289 | 46 | 48 | 267 |
| Ahtanum Creek | 730.83 | 125 | 18.09 | 0.826 | 80 | 134 | 39 | 7336 | 276 | -65 | 341 | -8 | 172 | 175 | -12 | 351 | 60 | 7 | 60 | 171 | 32 | 37 | 158 |
| Alpowa Creek | 720.74 | 6 | 17.37 | 0.840 | 100 | 127 | 37 | 7259 | 301 | -38 | 339 | 15 | 192 | 195 | 10 | 441 | 51 | 17 | 29 | 151 | 62 | 75 | 138 |
| Asotin Creek | 741.84 | 80 | 21.16 | 0.783 | 104 | 126 | 36 | 7379 | 306 | -35 | 341 | 17 | 197 | 201 | 12 | 429 | 47 | 18 | 26 | 140 | 65 | 78 | 127 |
| Bargamin Creek | 1087.47 | 0 | 11.56 | 0.834 | 19 | 165 | 42 | 7491 | 240 | -151 | 391 | -66 | 112 | 117 | -75 | 649 | 76 | 32 | 23 | 205 | 114 | 133 | 198 |
| Bear Creek | 1155.09 | 2 | 15.53 | 0.823 | 49 | 157 | 40 | 7614 | 271 | -113 | 384 | 84 | 142 | 149 | -47 | 501 | 58 | 28 | 22 | 156 | 99 | 117 | 131 |
| Bear Valley Creek | 969.82 | 0 | 14.16 | 0.791 | 3 | 173 | 42 | 7922 | 231 | -180 | 411 | -86 | 100 | 105 | -99 | 570 | 72 | 26 | 29 | 201 | 92 | 106 | 196 |
| Beech Creek | 1294.64 | 117 | 16.67 | 0.844 | 78 | 164 | 44 | 6912 | 297 | -75 | 372 | -1 | 165 | 171 | -7 | 346 | 40 | 14 | 27 | 114 | 54 | 66 | 100 |
| Belshaw Creek | 724.47 | 158 | 19.65 | 0.822 | 74 | 163 | 44 | 6808 | 290 | -78 | 368 | -4 | 160 | 165 | -11 | 361 | 42 | 15 | 27 | 121 | 55 | 67 | 107 |
| Big Creek | 706.51 | 76 | 17.15 | 0.833 | 30 | 170 | 41 | 8012 | 259 | -151 | 410 | -64 | 127 | 132 | -74 | 481 | 52 | 28 | 20 | 143 | 94 | 111 | 137 |
| Big White Salmon | 704.08 | 2 | 17.47 | 0.839 | 93 | 122 | 39 | 6603 | 275 | -35 | 310 | 15 | 179 | 179 | 10 | 863 | 160 | 9 | 74 | 451 | 47 | 47 | 424 |
| Black Canyon Creek | 278.11 | 0 | 15.57 | 0.860 | 64 | 164 | 44 | 6891 | 280 | -91 | 371 | -16 | 151 | 155 | -22 | 357 | 43 | 15 | 29 | 125 | 55 | 66 | 109 |
| Boulder Creek | 987.66 | 0 | 11.22 | 0.831 | 38 | 156 | 40 | 7664 | 258 | -128 | 386 | -49 | 132 | 137 | -60 | 670 | 87 | 22 | 34 | 242 | 88 | 106 | 234 |
| Bowman Creek | 318.13 | 15 | 15.11 | 0.822 | 84 | 129 | 39 | 6900 | 275 | -51 | 326 | 2 | 174 | 174 | -3 | 596 | 111 | 8 | 73 | 314 | 39 | 39 | 292 |
| Bridge Creek | 766.81 | 144 | 19.46 | 0.806 | 95 | 152 | 44 | 6667 | 299 | -46 | 345 | 17 | 180 | 184 | 13 | 320 | 40 | 12 | 30 | 111 | 46 | 56 | 95 |
| Buck Creek | 560.89 | 0 | 12.39 | 0.821 | 94 | 118 | 39 | 6307 | 268 | -28 | 296 | 21 | 176 | 176 | 15 | 1219 | 220 | 11 | 73 | 624 | 63 | 63 | 590 |
| Camp Creek | 275.65 | 13 | 17.4 | 0.847 | 42 | 156 | 42 | 6839 | 252 | -113 | 365 | -36 | 128 | 131 | -45 | 539 | 74 | 19 | 36 | 207 | 73 | 86 | 188 |
| Canyon Creek | 901.47 | 0 | 13.6 | 0.831 | 55 | 150 | 40 | 7374 | 270 | -99 | 369 | -31 | 146 | 152 | -38 | 767 | 89 | 31 | 27 | 249 | 115 | 134 | 235 |
| Catherine Creek | 1002.73 | 156 | 18.77 | 0.831 | 83 | 141 | 40 | 7144 | 291 | -59 | 350 | 0 | 173 | 177 | -7 | 445 | 51 | 16 | 28 | 147 | 63 | 79 | 134 |
| Chamberlain Creek | 1112.57 | 199 | 10.26 | 0.847 | 21 | 170 | 42 | 7635 | 245 | -155 | 400 | -67 | 114 | 120 | -76 | 617 | 73 | 31 | 23 | 197 | 108 | 127 | 190 |
| Clackamas River | 235.47 | 71 | 16.37 | 0.836 | 106 | 106 | 42 | 5081 | 255 | 5 | 250 | 48 | 171 | 172 | 44 | 1665 | 256 | 25 | 56 | 738 | 133 | 134 | 684 |
| Clear_Granite Creek | 796.13 | 113 | 15.25 | 0.847 | 44 | 163 | 42 | 7144 | 261 | -120 | 381 | -39 | 132 | 137 | -48 | 500 | 70 | 18 | 37 | 194 | 69 | 82 | 177 |
| Cowiche/ Crow Creek | 802.07 | 31 | 11.82 | 0.869 | 42 | 85 | 32 | 6224 | 194 | -68 | 262 | -28 | 122 | 124 | -33 | 1815 | 303 | 31 | 61 | 861 | 145 | 156 | 800 |
| Cowlitz River | 190.81 | 120 | 14.45 | 0.834 | 97 | 110 | 43 | 4995 | 246 | -5 | 251 | 40 | 162 | 162 | 36 | 1557 | 247 | 28 | 58 | 719 | 133 | 138 | 651 |
| Crooked Creek | 889.43 | 150 | 7.13 | 0.856 | 46 | 135 | 39 | 6886 | 248 | -95 | 343 | -33 | 134 | 136 | -40 | 691 | 90 | 21 | 38 | 264 | 85 | 99 | 246 |
| Crooked Fork Lochsa River | 1000.68 | 21 | 14.14 | 0.777 | 35 | 149 | 39 | 7521 | 253 | -121 | 374 | 66 | -15 | 134 | -58 | 477 | 56 | 29 | 21 | 148 | 102 | 119 | 121 |
| Crooked River | 945.39 | 174 | 15.67 | 0.841 | 32 | 161 | 41 | 7375 | 249 | -135 | 384 | -52 | 124 | 129 | -61 | 747 | 90 | 32 | 26 | 243 | 116 | 138 | 235 |
| Dead Canyon Creek | 334.94 | 129 | 15.74 | 0.831 | 84 | 129 | 39 | 6900 | 275 | -51 | 326 | 2 | 174 | 174 | -3 | 596 | 111 | 8 | 73 | 314 | 39 | 39 | 292 |
| Deer Creek | 725.64 | 76 | 13.81 | 0.828 | 64 | 164 | 44 | 6891 | 280 | -91 | 371 | -16 | 151 | 155 | -22 | 357 | 43 | 15 | 29 | 125 | 55 | 66 | 109 |
| Deschutes River | 625.17 | 80 | 17.62 | 0.833 | 97 | 142 | 42 | 6820 | 297 | -41 | 338 | 16 | 183 | 186 | 12 | 389 | 61 | 8 | 53 | 175 | 38 | 40 | 161 |
| Dillacort Creek | 293.25 | 35 | 15.31 | 0.837 | 92 | 125 | 39 | 6836 | 279 | -41 | 320 | 11 | 180 | 180 | 6 | 643 | 121 | 7 | 74 | 339 | 38 | 38 | 317 |
| EF Salmon River | 289.76 | 162 | 12.75 | 0.822 | 87 | 118 | 40 | 6192 | 261 | -32 | 293 | 16 | 168 | 168 | 11 | 1271 | 223 | 13 | 69 | 632 | 73 | 73 | 598 |
| Eagle Creek | 1395.98 | 1 | 11.81 | 0.845 | 6 | 169 | 41 | 8162 | 232 | -179 | 411 | -85 | 72 | 111 | -98 | 445 | 50 | 26 | 21 | 138 | 87 | 102 | 136 |
| Elochoman River | 768.76 | 87 | 14.82 | 0.803 | 92 | 102 | 43 | 4671 | 228 | -4 | 232 | 40 | 152 | 154 | 36 | 2038 | 324 | 32 | 61 | 948 | 147 | 164 | 882 |
| Entiat River | 65.84 | 175 | 16.67 | 0.841 | 77 | 122 | 33 | 8365 | 281 | -82 | 363 | -24 | 179 | 183 | -31 | 377 | 64 | 10 | 55 | 177 | 41 | 47 | 160 |
| Fifteenmile Creek | 352.92 | 99 | 17.79 | 0.825 | 89 | 138 | 41 | 6782 | 287 | -47 | 334 | 9 | 175 | 178 | 4 | 428 | 70 | 8 | 57 | 198 | 39 | 40 | 184 |
| Fish Creek | 926.57 | 0 | 18.01 | 0.830 | 52 | 153 | 40 | 7441 | 270 | -105 | 375 | -34 | 144 | 150 | -41 | 707 | 81 | 30 | 25 | 225 | 111 | 130 | 212 |
| Foster | 378.57 | 97 | 17.1 | 0.840 | 113 | 118 | 44 | 5108 | 273 | 6 | 267 | 54 | 180 | 180 | 51 | 1145 | 179 | 14 | 60 | 523 | 80 | 80 | 486 |
| Fox Creek | 700.56 | 240 | 15.63 | 0.830 | 55 | 158 | 44 | 6637 | 265 | -94 | 359 | -20 | 139 | 143 | -27 | 435 | 54 | 17 | 30 | 154 | 64 | 76 | 136 |
| Gedney Creek | 916.8 | 7 | 15.68 | 0.850 | 45 | 157 | 41 | 7492 | 265 | -117 | 382 | -42 | 138 | 143 | -49 | 650 | 73 | 31 | 23 | 200 | 111 | 130 | 189 |
| George Creek | 749.81 | 0 | 16.75 | 0.842 | 91 | 128 | 37 | 7262 | 293 | -48 | 341 | 6 | 183 | 186 | 1 | 469 | 51 | 19 | 25 | 150 | 71 | 85 | 137 |

Table A3. Continued.

| **Population** | **Migration Distance** | **Eleva-tion** | **Water Temp** | **Heat Load Index** | **Annual Mean Temp** | **Mean Diurnal Range** | **Isother-mality** | **Temp Season-ality** | **Min Temp Warmest Month** | **Min Temp Coldest Month** | **Temp Annual Range** | **Mean Temp Wettest Quarter** | **Mean Temp Driest Quarter** | **Mean Temp Warmest Quarter** | **Mean Temp Coldest Quarter** | **Annual Precip** | **Precip Wettest Month** | **Precip Driest Month** | **Precip Season-ality** | **Precip Wettest Quarter** | **Precip Driest Quarter** | **Precip Warmest Quarter** | **Precip Coldest Quarter** |
| --- | --- | --- | --- | --- | --- | --- | --- | --- | --- | --- | --- | --- | --- | --- | --- | --- | --- | --- | --- | --- | --- | --- | --- |
| Germany Creek | 89.37 | 70 | 16.1 | 0.809 | 95 | 104 | 43 | 4798 | 236 | -3 | 239 | 40 | 156 | 158 | 37 | 1775 | 279 | 29 | 60 | 823 | 136 | 148 | 758 |
| Gumboot and Mahogany Creeks | 909.59 | 37 | 11.11 | 0.822 | 37 | 150 | 40 | 7469 | 251 | -123 | 374 | -49 | 130 | 133 | -59 | 586 | 70 | 22 | 29 | 200 | 86 | 105 | 190 |
| Hayden Creek | 1274.09 | 64 | 12.69 | 0.831 | 38 | 166 | 39 | 8495 | 270 | -148 | 418 | 123 | -52 | 147 | -72 | 271 | 43 | 10 | 44 | 109 | 38 | 97 | 40 |
| Icicle Creek | 788.09 | 0 | 14.82 | 0.824 | 40 | 108 | 34 | 7031 | 219 | -92 | 311 | -41 | 131 | 131 | -47 | 1142 | 199 | 22 | 63 | 563 | 98 | 98 | 519 |
| Indian Creek | 787.65 | 2 | 16.3 | 0.839 | 93 | 122 | 39 | 6603 | 275 | -35 | 310 | 15 | 179 | 179 | 10 | 863 | 160 | 9 | 74 | 451 | 47 | 47 | 424 |
| Iskuulpa Creek | 278.22 | 92 | 18.59 | 0.803 | 93 | 130 | 38 | 7071 | 297 | -43 | 340 | 11 | 183 | 187 | 6 | 574 | 77 | 13 | 44 | 227 | 58 | 67 | 208 |
| Joseph Creek | 688.28 | 129 | 21.14 | 0.865 | 82 | 134 | 38 | 7225 | 286 | -61 | 347 | -3 | 173 | 176 | -9 | 500 | 54 | 21 | 25 | 159 | 77 | 93 | 145 |
| Kalama River | 113.62 | 23 | 16.35 | 0.814 | 106 | 108 | 43 | 5002 | 252 | 4 | 248 | 48 | 169 | 171 | 44 | 1398 | 221 | 24 | 58 | 638 | 116 | 122 | 586 |
| Lapwai Creek | 779.07 | 210 | 16.66 | 0.840 | 82 | 128 | 37 | 7199 | 284 | -57 | 341 | -2 | 173 | 177 | -7 | 601 | 65 | 25 | 26 | 192 | 91 | 106 | 178 |
| Lewis River | 167.08 | 72 | 13.83 | 0.811 | 101 | 109 | 42 | 5100 | 253 | -2 | 255 | 43 | 167 | 168 | 39 | 1835 | 295 | 27 | 59 | 845 | 142 | 148 | 785 |
| Lightning Creek | 1009.37 | 10 | 16.27 | 0.894 | 56 | 145 | 39 | 7345 | 266 | -97 | 363 | -29 | 148 | 151 | -37 | 555 | 59 | 23 | 24 | 170 | 88 | 107 | 157 |
| Little Clear-water River | 838.14 | 0 | 13.82 | 0.805 | 34 | 165 | 41 | 7633 | 257 | -136 | 393 | 68 | 127 | 133 | -63 | 528 | 58 | 30 | 21 | 158 | 104 | 122 | 145 |
| Little Klickitat River | 316.53 | 8 | 18.01 | 0.824 | 84 | 129 | 39 | 6900 | 275 | -51 | 326 | 2 | 174 | 174 | -3 | 596 | 111 | 8 | 73 | 314 | 39 | 39 | 292 |
| Little Naches River | 820.39 | 36 | 11.2 | 0.855 | 42 | 85 | 32 | 6224 | 194 | -68 | 262 | -28 | 122 | 124 | -33 | 1815 | 303 | 31 | 61 | 861 | 145 | 156 | 800 |
| Little Rattlesnake Creek | 780.46 | 43 | 10.1 | 0.838 | 44 | 99 | 34 | 6444 | 208 | -75 | 283 | -29 | 127 | 129 | -34 | 1282 | 217 | 22 | 63 | 623 | 104 | 107 | 578 |
| Little and Big Bear Creeks | 782.58 | 130 | 18.12 | 0.817 | 90 | 128 | 37 | 7239 | 292 | -49 | 341 | 4 | 181 | 184 | -1 | 578 | 67 | 22 | 30 | 200 | 80 | 94 | 185 |
| Lolo Creek | 880.85 | 163 | 12.61 | 0.835 | 54 | 145 | 39 | 7316 | 267 | -96 | 363 | -30 | 145 | 151 | -38 | 874 | 108 | 31 | 32 | 304 | 119 | 136 | 290 |
| Mad Creek | 82.72 | 120 | 15.17 | 0.838 | 88 | 106 | 42 | 5063 | 239 | -10 | 249 | 33 | 154 | 156 | 28 | 2182 | 351 | 26 | 61 | 1003 | 148 | 151 | 930 |
| MF Hood River | 289.64 | 0 | 12.75 | 0.816 | 87 | 118 | 40 | 6192 | 261 | -32 | 293 | 16 | 168 | 168 | 11 | 1271 | 223 | 13 | 69 | 632 | 73 | 73 | 598 |
| Mill Creek | 413.79 | 0 | 14.79 | 0.835 | 92 | 102 | 43 | 4671 | 228 | -4 | 232 | 40 | 152 | 154 | 36 | 2038 | 324 | 32 | 61 | 948 | 147 | 164 | 882 |
| Mission Creek | 773.55 | 91 | 17.4 | 0.811 | 67 | 130 | 38 | 7082 | 268 | -73 | 341 | -15 | 157 | 160 | -21 | 592 | 63 | 25 | 25 | 184 | 92 | 109 | 169 |
| Moose Creek | 959.78 | 0 | 13.56 | 0.839 | 41 | 155 | 40 | 7540 | 261 | -119 | 380 | 74 | 135 | 140 | -53 | 513 | 59 | 29 | 22 | 158 | 103 | 120 | 135 |
| Morgan Creek | 1321.36 | 0 | 13.04 | 0.829 | 17 | 169 | 40 | 8182 | 246 | -167 | 413 | 55 | -36 | 122 | -88 | 381 | 47 | 23 | 22 | 120 | 80 | 107 | 91 |
| MS John Day River | 578.87 | 175 | 19.91 | 0.838 | 77 | 164 | 44 | 6743 | 292 | -73 | 365 | -1 | 162 | 167 | -6 | 353 | 42 | 14 | 29 | 121 | 52 | 65 | 106 |
| Murderer's Creek | 708.94 | 14 | 18.46 | 0.832 | 64 | 164 | 44 | 6891 | 280 | -91 | 371 | -16 | 151 | 155 | -22 | 357 | 43 | 15 | 29 | 125 | 55 | 66 | 109 |
| Nason Creek | 937.05 | 0 | 15.93 | 0.851 | 59 | 122 | 37 | 7245 | 248 | -81 | 329 | -27 | 152 | 152 | -33 | 1005 | 186 | 17 | 71 | 527 | 76 | 76 | 485 |
| Newsome Creek | 829.15 | 36 | 14.67 | 0.849 | 36 | 158 | 41 | 7340 | 251 | -128 | 379 | -48 | 127 | 132 | -56 | 756 | 90 | 32 | 26 | 243 | 118 | 140 | 234 |
| NF John Day River / Desolation Creek | 275.65 | 44 | 17.89 | 0.864 | 58 | 157 | 43 | 6706 | 268 | -93 | 361 | -20 | 142 | 146 | -27 | 484 | 62 | 17 | 34 | 179 | 65 | 78 | 160 |
| NF Salmon River | 1188.66 | 0 | 14.09 | 0.832 | 41 | 168 | 40 | 8193 | 271 | -140 | 411 | 82 | -45 | 146 | -65 | 345 | 48 | 18 | 31 | 122 | 64 | 105 | 69 |
| Nile Creek | 778.31 | 78 | 12.37 | 0.860 | 50 | 101 | 35 | 6521 | 215 | -72 | 287 | -25 | 134 | 135 | -30 | 1222 | 209 | 21 | 63 | 595 | 99 | 103 | 552 |
| Ohara Creek | 904.65 | 0 | 14.42 | 0.841 | 58 | 152 | 40 | 7414 | 275 | -98 | 373 | -28 | 150 | 156 | -35 | 737 | 82 | 31 | 25 | 231 | 113 | 134 | 216 |
| Omak Creek | 899.14 | 65 | 17.98 | 0.848 | 87 | 130 | 34 | 8705 | 296 | -84 | 380 | -19 | 191 | 197 | -27 | 312 | 43 | 14 | 31 | 114 | 48 | 59 | 107 |
| Pahsimeroi River | 1298.26 | 3 | 13.79 | 0.857 | 46 | 168 | 39 | 8603 | 281 | -143 | 424 | 131 | -44 | 156 | -67 | 279 | 40 | 13 | 32 | 101 | 49 | 89 | 55 |
| Panther Creek | 1190.06 | 89 | 12.48 | 0.836 | 22 | 170 | 41 | 8093 | 251 | -160 | 411 | 60 | -29 | 126 | -83 | 405 | 50 | 25 | 22 | 130 | 86 | 111 | 96 |
| Pistol Creek | 1243.41 | 153 | 14.2 | 0.802 | 21 | 174 | 42 | 8028 | 251 | -162 | 413 | -72 | 118 | 124 | -83 | 520 | 61 | 26 | 24 | 171 | 91 | 106 | 165 |
| Potlatch River | 820.78 | 118 | 18.22 | 0.828 | 67 | 133 | 38 | 7172 | 271 | -75 | 346 | -17 | 157 | 161 | -23 | 810 | 106 | 27 | 36 | 302 | 104 | 117 | 289 |
| Quartz Creek | 1249.41 | 44 | 13.21 | 0.815 | 78 | 123 | 37 | 7018 | 268 | -57 | 325 | -4 | 168 | 170 | -9 | 453 | 77 | 9 | 61 | 220 | 41 | 43 | 199 |
| Rapid River | 402.09 | 0 | 14.75 | 0.830 | 21 | 174 | 42 | 8028 | 251 | -162 | 413 | -72 | 118 | 124 | -83 | 520 | 61 | 26 | 24 | 171 | 91 | 106 | 165 |
| Rattlesnake Creek | 277.84 | 2 | 17.47 | 0.839 | 93 | 122 | 39 | 6603 | 275 | -35 | 310 | 15 | 179 | 179 | 10 | 863 | 160 | 9 | 74 | 451 | 47 | 47 | 424 |
| Satus Creek | 698.95 | 74 | 15.86 | 0.821 | 90 | 134 | 38 | 7452 | 289 | -56 | 345 | 0 | 183 | 187 | -4 | 312 | 54 | 6 | 59 | 152 | 30 | 32 | 139 |
| Secesh River | 1104.87 | 159 | 10.67 | 0.839 | 27 | 166 | 42 | 7479 | 250 | -141 | 391 | -57 | 120 | 125 | -67 | 679 | 86 | 26 | 30 | 235 | 100 | 118 | 225 |
| Selway River | 929.33 | 0 | 9.02 | 0.875 | 45 | 157 | 41 | 7492 | 265 | -117 | 382 | -42 | 138 | 143 | -49 | 650 | 73 | 31 | 23 | 200 | 111 | 130 | 189 |

Table A3. Continued.

| **Population** | **Migration Distance** | **Eleva-tion** | **Water Temp** | **Heat Load Index** | **Annual Mean Temp** | **Mean Diurnal Range** | **Isother-mality** | **Temp Season-ality** | **Min Temp Warmest Month** | **Min Temp Coldest Month** | **Temp Annual Range** | **Mean Temp Wettest Quarter** | **Mean Temp Driest Quarter** | **Mean Temp Warmest Quarter** | **Mean Temp Coldest Quarter** | **Annual Precip** | **Precip Wettest Month** | **Precip Driest Month** | **Precip Season-ality** | **Precip Wettest Quarter** | **Precip Driest Quarter** | **Precip Warmest Quarter** | **Precip Coldest Quarter** |
| --- | --- | --- | --- | --- | --- | --- | --- | --- | --- | --- | --- | --- | --- | --- | --- | --- | --- | --- | --- | --- | --- | --- | --- |
| SF John Day River | 288.41 | 0 | 18.71 | 0.848 | 77 | 164 | 44 | 6743 | 292 | -73 | 365 | -1 | 162 | 167 | -6 | 353 | 42 | 14 | 29 | 121 | 52 | 65 | 106 |
| SF Santiam River | 350.85 | 82 | 18.19 | 0.831 | 112 | 120 | 44 | 5167 | 275 | 6 | 269 | 53 | 179 | 180 | 50 | 1112 | 183 | 12 | 65 | 528 | 69 | 69 | 499 |
| Sheep Creek | 877.09 | 0 | 20.97 | 0.833 | 54 | 147 | 39 | 7462 | 267 | -101 | 368 | -31 | 148 | 152 | -40 | 593 | 67 | 23 | 27 | 191 | 88 | 108 | 180 |
| Simcoe Creek | 740.6 | 95 | 15.23 | 0.828 | 73 | 126 | 38 | 6988 | 262 | -63 | 325 | -10 | 161 | 164 | -14 | 570 | 102 | 10 | 67 | 289 | 46 | 48 | 267 |
| Skamania Stock | 235.47 | 71 | 16.37 | 0.836 | 106 | 106 | 42 | 5081 | 255 | 5 | 250 | 48 | 171 | 172 | 44 | 1665 | 256 | 25 | 56 | 738 | 133 | 134 | 684 |
| Slate Creek | 930.78 | 187 | 14.62 | 0.786 | 33 | 154 | 41 | 7199 | 245 | -126 | 371 | -48 | 123 | 127 | -57 | 719 | 84 | 29 | 27 | 231 | 109 | 131 | 220 |
| Snyder Creek | 307.89 | 0 | 17.29 | 0.854 | 87 | 131 | 39 | 6863 | 280 | -49 | 329 | 5 | 176 | 176 | 0 | 526 | 97 | 7 | 71 | 272 | 36 | 36 | 253 |
| Summit Creek | 343.41 | 24 | 13.85 | 0.851 | 84 | 129 | 39 | 6900 | 275 | -51 | 326 | 2 | 174 | 174 | -3 | 596 | 111 | 8 | 73 | 314 | 39 | 39 | 292 |
| Surveyors Creek | 379.86 | 0 | 10.96 | 0.825 | 55 | 107 | 36 | 6403 | 226 | -65 | 291 | -18 | 139 | 139 | -23 | 1276 | 222 | 18 | 67 | 638 | 89 | 89 | 595 |
| Swale and Wheeler Creeks | 315.45 | 176 | 18.22 | 0.823 | 87 | 131 | 39 | 6863 | 280 | -49 | 329 | 5 | 176 | 176 | 0 | 526 | 97 | 7 | 71 | 272 | 36 | 36 | 253 |
| Sweetwater Creek | 771.11 | 166 | 15.26 | 0.840 | 92 | 127 | 37 | 7310 | 294 | -47 | 341 | 7 | 185 | 188 | 2 | 476 | 51 | 21 | 24 | 145 | 76 | 90 | 132 |
| Teanaway River | 835.03 | 119 | 14.05 | 0.849 | 64 | 117 | 36 | 7029 | 246 | -72 | 318 | -19 | 154 | 154 | -24 | 873 | 154 | 16 | 65 | 434 | 71 | 71 | 399 |
| Tenmile Creek | 939.09 | 153 | 11.21 | 0.887 | 31 | 158 | 41 | 7260 | 246 | -132 | 378 | -51 | 122 | 127 | -59 | 753 | 89 | 31 | 26 | 243 | 116 | 138 | 233 |
| ThreeLinks Creek | 940.49 | 0 | 14.92 | 0.828 | 45 | 158 | 41 | 7544 | 266 | -118 | 384 | 79 | 138 | 144 | -50 | 578 | 63 | 30 | 21 | 172 | 106 | 125 | 162 |
| Toppenish Creek | 737.48 | 7 | 15.53 | 0.854 | 66 | 120 | 38 | 6864 | 250 | -65 | 315 | -14 | 156 | 156 | -19 | 673 | 120 | 11 | 67 | 342 | 53 | 53 | 316 |
| Touchet River | 528.4 | 85 | 21.88 | 0.827 | 115 | 131 | 37 | 7518 | 316 | -30 | 346 | 25 | 208 | 214 | 20 | 290 | 39 | 8 | 39 | 113 | 34 | 38 | 101 |
| Trout Creek | 353.21 | 121 | 15.26 | 0.831 | 76 | 123 | 38 | 6755 | 262 | -54 | 316 | -4 | 164 | 164 | -9 | 888 | 164 | 11 | 73 | 466 | 55 | 55 | 436 |
| Tucannon River | 673.9 | 240 | 14.59 | 0.820 | 47 | 131 | 38 | 6861 | 246 | -91 | 337 | -32 | 135 | 136 | -38 | 692 | 91 | 21 | 38 | 266 | 84 | 97 | 248 |
| Warm Springs River | 900.24 | 40 | 16.15 | 0.786 | 83 | 143 | 43 | 6387 | 278 | -48 | 326 | 9 | 165 | 168 | 5 | 572 | 87 | 12 | 52 | 255 | 57 | 60 | 233 |
| Washougal River Still Creek | 474.42 | 0 | 12.24 | 0.858 | 66 | 100 | 39 | 5426 | 220 | -35 | 255 | 8 | 137 | 139 | 3 | 2170 | 354 | 26 | 62 | 1013 | 145 | 153 | 956 |
| Webb Creek | 259.96 | 28 | 16.83 | 0.863 | 67 | 130 | 38 | 7082 | 268 | -73 | 341 | -15 | 157 | 160 | -21 | 592 | 63 | 25 | 25 | 184 | 92 | 109 | 169 |
| Wenaha River | 769.73 | 171 | 15.91 | 0.830 | 72 | 143 | 40 | 7128 | 281 | -73 | 354 | -12 | 161 | 165 | -18 | 546 | 64 | 20 | 30 | 191 | 77 | 90 | 175 |
| WF Hood River | 286.09 | 48 | 13.17 | 0.803 | 87 | 118 | 40 | 6192 | 261 | -32 | 293 | 16 | 168 | 168 | 11 | 1271 | 223 | 13 | 69 | 632 | 73 | 73 | 598 |
| White Creek | 347.21 | 223 | 14.5 | 0.806 | 72 | 122 | 38 | 6786 | 258 | -59 | 317 | -8 | 161 | 161 | -13 | 740 | 136 | 10 | 72 | 386 | 50 | 50 | 359 |
| White Bird Creek | 852.52 | 0 | 16.23 | 0.866 | 84 | 138 | 38 | 7366 | 292 | -62 | 354 | 115 | 177 | 182 | -7 | 557 | 68 | 26 | 26 | 184 | 94 | 114 | 131 |
| White Cap Creek | 996.51 | 129 | 12.45 | 0.830 | 44 | 161 | 41 | 7610 | 266 | -122 | 388 | 79 | 137 | 143 | -53 | 494 | 57 | 29 | 21 | 153 | 100 | 118 | 130 |
| Wiley Creek | 407.68 | 67 | 12.93 | 0.820 | 106 | 119 | 44 | 5025 | 267 | 2 | 265 | 49 | 173 | 173 | 45 | 1435 | 227 | 18 | 59 | 653 | 103 | 103 | 595 |

Table A4. Minor allele frequency (MAF) for all steelhead collections genotyped at all 13 markers. The table includes average MAF for all collections and by lineage. Fixed alleles are outlined in red and averaged MAF for markers 8-12 are highlighted gray.

| **MAF Summary** | **1** | **2** | **3** | **4** | **5** | **6** | **7** | **8** | **9** | **10** | **11** | **12** | **13** |
| --- | --- | --- | --- | --- | --- | --- | --- | --- | --- | --- | --- | --- | --- |
| All collection averages | 0.190 | 0.318 | 0.287 | 0.156 | 0.323 | 0.145 | 0.134 | 0.281 | 0.314 | 0.259 | 0.292 | 0.280 | 0.253 |
| Coastal collection averages | 0.386 | 0.461 | 0.405 | 0.213 | 0.527 | 0.220 | 0.206 | 0.246 | 0.286 | 0.233 | 0.281 | 0.256 | 0.306 |
| Inland collection averages | 0.022 | 0.134 | 0.117 | 0.038 | 0.122 | 0.017 | 0.017 | 0.223 | 0.241 | 0.196 | 0.218 | 0.217 | 0.188 |
| **MAF coastal collections** | **1** | **2** | **3** | **4** | **5** | **6** | **7** | **8** | **9** | **10** | **11** | **12** | **13** |
| Abernathy FTC | 0.661 | 0.733 | 0.494 | 0.122 | 0.800 | 0.111 | 0.111 | 0.206 | 0.261 | 0.172 | 0.361 | 0.217 | 0.172 |
| Buck Creek | 0.354 | 0.486 | 0.465 | 0.361 | 0.500 | 0.354 | 0.354 | 0.472 | 0.486 | 0.389 | 0.493 | 0.486 | 0.403 |
| Clackamas River | 0.139 | 0.307 | 0.278 | 0.046 | 0.198 | 0.075 | 0.044 | 0.052 | 0.108 | 0.046 | 0.067 | 0.054 | 0.098 |
| Cowlitz River | 0.104 | 0.108 | 0.063 | 0.000 | 0.188 | 0.000 | 0.000 | 0.000 | 0.000 | 0.000 | 0.000 | 0.000 | 0.071 |
| Eagle Creek | 0.354 | 0.415 | 0.341 | 0.000 | 0.451 | 0.000 | 0.000 | 0.049 | 0.073 | 0.012 | 0.098 | 0.061 | 0.171 |
| Elochoman River | 0.171 | 0.171 | 0.122 | 0.000 | 0.463 | 0.000 | 0.000 | 0.000 | 0.024 | 0.037 | 0.037 | 0.024 | 0.195 |
| Germany Creek | 0.375 | 0.417 | 0.361 | 0.028 | 0.569 | 0.028 | 0.028 | 0.111 | 0.139 | 0.097 | 0.181 | 0.153 | 0.139 |
| Gray River | 0.135 | 0.135 | 0.077 | 0.000 | 0.519 | 0.000 | 0.000 | 0.019 | 0.019 | 0.000 | 0.038 | 0.019 | 0.077 |
| Lewis River | 0.253 | 0.253 | 0.138 | 0.000 | 0.305 | 0.000 | 0.000 | 0.000 | 0.017 | 0.029 | 0.029 | 0.017 | 0.132 |
| Mad Creek | 0.621 | 0.672 | 0.672 | 0.586 | 0.672 | 0.552 | 0.552 | 0.569 | 0.621 | 0.672 | 0.603 | 0.534 | 0.603 |
| Rattlesnake Creek | 0.348 | 0.667 | 0.662 | 0.172 | 0.784 | 0.123 | 0.113 | 0.328 | 0.387 | 0.137 | 0.358 | 0.387 | 0.534 |
| Skamania Stock | 0.990 | 1.000 | 1.000 | 1.000 | 1.000 | 1.000 | 1.000 | 0.993 | 1.000 | 1.000 | 1.000 | 0.997 | 0.910 |
| Washougal_Still Creek | 0.593 | 0.620 | 0.537 | 0.398 | 0.676 | 0.398 | 0.398 | 0.407 | 0.444 | 0.417 | 0.435 | 0.417 | 0.463 |
| WF Hood River | 0.323 | 0.554 | 0.497 | 0.253 | 0.366 | 0.425 | 0.250 | 0.234 | 0.465 | 0.242 | 0.255 | 0.234 | 0.288 |
| Wiley Creek | 0.375 | 0.383 | 0.359 | 0.234 | 0.414 | 0.234 | 0.234 | 0.242 | 0.250 | 0.242 | 0.266 | 0.242 | 0.328 |

Table A4. Continued.

| **MAF inland collections** | **1** | **2** | **3** | **4** | **5** | **6** | **7** | **8** | **9** | **10** | **11** | **12** | **13** |
| --- | --- | --- | --- | --- | --- | --- | --- | --- | --- | --- | --- | --- | --- |
| Ahtanum Creek | 0.006 | 0.184 | 0.184 | 0.000 | 0.203 | 0.000 | 0.000 | 0.222 | 0.241 | 0.114 | 0.222 | 0.209 | 0.165 |
| Belshaw Creek | 0.000 | 0.300 | 0.000 | 0.300 | 0.300 | 0.000 | 0.000 | 0.280 | 0.300 | 0.000 | 0.000 | 0.300 | 0.000 |
| Black Canyon Creek | 0.000 | 0.023 | 0.012 | 0.000 | 0.035 | 0.000 | 0.000 | 0.035 | 0.035 | 0.035 | 0.023 | 0.047 | 0.070 |
| Camp Creek | 0.000 | 0.013 | 0.013 | 0.000 | 0.013 | 0.000 | 0.000 | 0.038 | 0.050 | 0.050 | 0.050 | 0.050 | 0.075 |
| Catherine Creek | 0.007 | 0.030 | 0.026 | 0.004 | 0.046 | 0.000 | 0.002 | 0.108 | 0.112 | 0.099 | 0.110 | 0.108 | 0.117 |
| Crooked Creek | 0.048 | 0.013 | 0.013 | 0.013 | 0.013 | 0.013 | 0.013 | 0.083 | 0.096 | 0.088 | 0.092 | 0.075 | 0.100 |
| Deschutes River | 0.027 | 0.163 | 0.158 | 0.005 | 0.136 | 0.005 | 0.005 | 0.299 | 0.321 | 0.315 | 0.321 | 0.288 | 0.277 |
| Entiat River | 0.028 | 0.111 | 0.111 | 0.014 | 0.111 | 0.014 | 0.014 | 0.361 | 0.389 | 0.333 | 0.389 | 0.361 | 0.319 |
| Fifteenmile Creek | 0.066 | 0.116 | 0.070 | 0.029 | 0.165 | 0.029 | 0.029 | 0.169 | 0.186 | 0.174 | 0.169 | 0.153 | 0.227 |
| Grande Ronde River | 0.000 | 0.021 | 0.021 | 0.000 | 0.043 | 0.000 | 0.000 | 0.106 | 0.117 | 0.106 | 0.106 | 0.138 | 0.138 |
| Icicle Creek | 0.075 | 0.175 | 0.175 | 0.075 | 0.175 | 0.075 | 0.075 | 0.525 | 0.575 | 0.475 | 0.575 | 0.500 | 0.500 |
| Iskuulpa_Desolation | 0.006 | 0.032 | 0.019 | 0.000 | 0.045 | 0.000 | 0.000 | 0.065 | 0.084 | 0.058 | 0.071 | 0.071 | 0.091 |
| MS John Day River | 0.005 | 0.022 | 0.019 | 0.002 | 0.024 | 0.000 | 0.000 | 0.071 | 0.073 | 0.066 | 0.065 | 0.069 | 0.088 |
| NF John Day River | 0.003 | 0.013 | 0.013 | 0.000 | 0.036 | 0.000 | 0.000 | 0.039 | 0.042 | 0.032 | 0.039 | 0.042 | 0.058 |
| SF John Day River | 0.004 | 0.018 | 0.018 | 0.000 | 0.018 | 0.000 | 0.000 | 0.068 | 0.072 | 0.063 | 0.068 | 0.066 | 0.066 |
| Lightning Creek | 0.000 | 0.020 | 0.020 | 0.000 | 0.020 | 0.000 | 0.000 | 0.158 | 0.158 | 0.158 | 0.158 | 0.158 | 0.118 |
| Little Rattlesnake | 0.008 | 0.133 | 0.117 | 0.016 | 0.125 | 0.008 | 0.008 | 0.125 | 0.133 | 0.047 | 0.125 | 0.125 | 0.125 |
| Nason Creek | 0.063 | 0.188 | 0.188 | 0.094 | 0.188 | 0.063 | 0.063 | 0.719 | 0.750 | 0.719 | 0.750 | 0.656 | 0.500 |
| Nile Creek | 0.051 | 0.165 | 0.148 | 0.040 | 0.165 | 0.023 | 0.023 | 0.193 | 0.216 | 0.091 | 0.182 | 0.193 | 0.142 |
| Omak Creek | 0.013 | 0.067 | 0.058 | 0.000 | 0.071 | 0.000 | 0.000 | 0.518 | 0.554 | 0.513 | 0.540 | 0.460 | 0.491 |
| Quartz Creek | 0.011 | 0.038 | 0.016 | 0.022 | 0.038 | 0.000 | 0.000 | 0.043 | 0.043 | 0.016 | 0.022 | 0.043 | 0.097 |
| Surveyors Creek | 0.017 | 0.950 | 0.950 | 0.367 | 0.633 | 0.200 | 0.183 | 0.950 | 0.950 | 0.950 | 0.917 | 0.833 | 0.650 |
| Teanaway River | 0.058 | 0.192 | 0.173 | 0.000 | 0.192 | 0.000 | 0.000 | 0.192 | 0.192 | 0.096 | 0.135 | 0.173 | 0.115 |
| Toppenish Creek | 0.004 | 0.018 | 0.017 | 0.000 | 0.014 | 0.000 | 0.000 | 0.013 | 0.025 | 0.007 | 0.014 | 0.012 | 0.014 |
| Warm Springs River | 0.044 | 0.431 | 0.438 | 0.000 | 0.300 | 0.000 | 0.000 | 0.363 | 0.425 | 0.425 | 0.419 | 0.381 | 0.256 |
| Wenaha River | 0.040 | 0.060 | 0.060 | 0.020 | 0.060 | 0.020 | 0.020 | 0.060 | 0.120 | 0.060 | 0.120 | 0.120 | 0.100 |

Figure A1. Relationship between candidate allele frequency (markers 2,3,6,9) and each environmental variable that was significant in RDA results from 113 steelhead collections in the Columbia River. The geographic region of each population is represented by shapes.


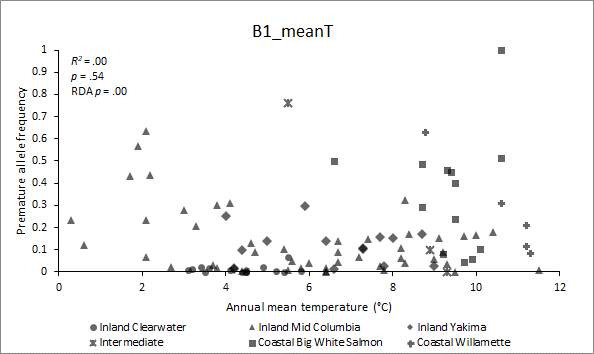


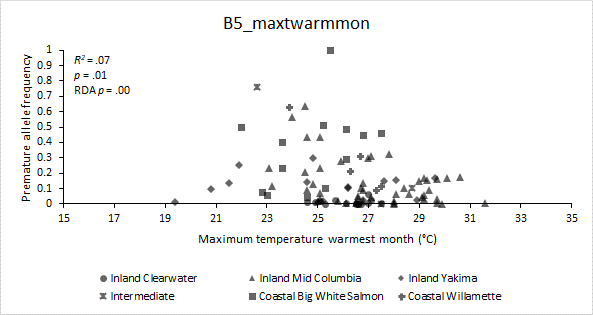


Figure A1. Continued.
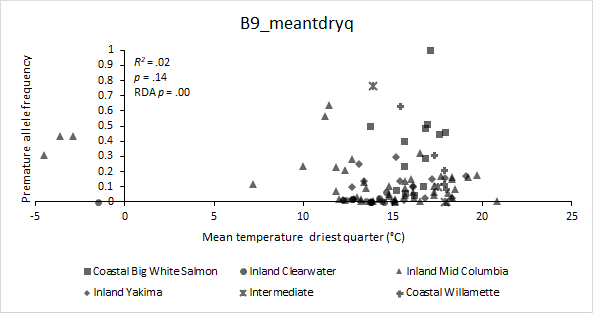


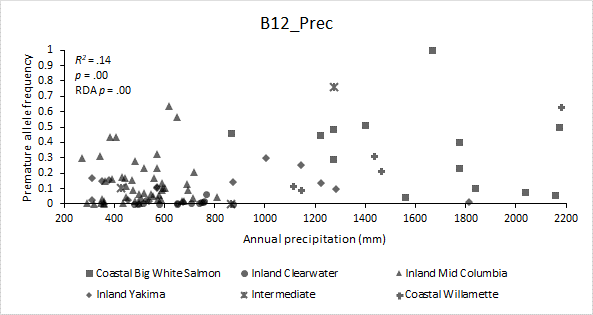


Figure A1. Continued.


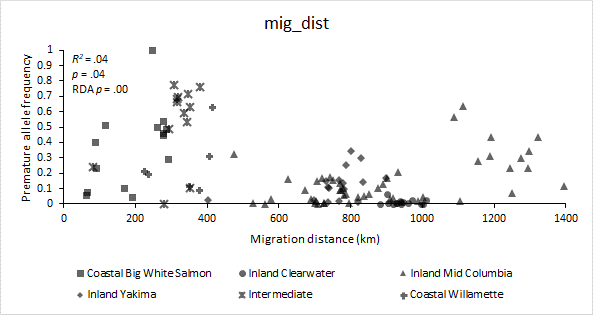


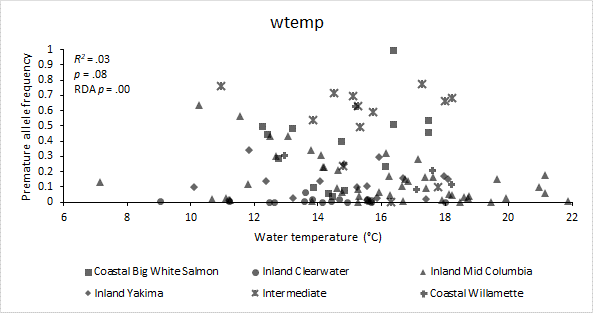

Supplement: Supplementary file 1 — Appendix S1 [file ECE3-10-9486-s001.docx]
